# DNA Damage Response and Spindle Assembly Checkpoint Function throughout the Cell Cycle to Ensure Genomic Integrity

**DOI:** 10.1371/journal.pgen.1005150

**Published:** 2015-04-21

**Authors:** Katherine S. Lawrence, Thinh Chau, JoAnne Engebrecht

**Affiliations:** Department of Molecular and Cellular Biology; Biochemistry, Molecular Cellular and Developmental Biology Graduate Group, University of California, Davis, Davis, California, United States of America; Imperial College, UNITED KINGDOM

## Abstract

Errors in replication or segregation lead to DNA damage, mutations, and aneuploidies. Consequently, cells monitor these events and delay progression through the cell cycle so repair precedes division. The DNA damage response (DDR), which monitors DNA integrity, and the spindle assembly checkpoint (SAC), which responds to defects in spindle attachment/tension during metaphase of mitosis and meiosis, are critical for preventing genome instability. Here we show that the DDR and SAC function together throughout the cell cycle to ensure genome integrity in *C*. *elegans* germ cells. Metaphase defects result in enrichment of SAC and DDR components to chromatin, and both SAC and DDR are required for metaphase delays. During persistent metaphase arrest following establishment of bi-oriented chromosomes, stability of the metaphase plate is compromised in the absence of DDR kinases ATR or CHK1 or SAC components, MAD1/MAD2, suggesting SAC functions in metaphase beyond its interactions with APC activator CDC20. In response to DNA damage, MAD2 and the histone variant CENPA become enriched at the nuclear periphery in a DDR-dependent manner. Further, depletion of either MAD1 or CENPA results in loss of peripherally associated damaged DNA. In contrast to a SAC-insensitive CDC20 mutant, germ cells deficient for SAC or CENPA cannot efficiently repair DNA damage, suggesting that SAC mediates DNA repair through CENPA interactions with the nuclear periphery. We also show that replication perturbations result in relocalization of MAD1/MAD2 in human cells, suggesting that the role of SAC in DNA repair is conserved.

## Author Summary

Checkpoints are surveillance pathways that monitor and correct cellular errors to ensure that the genome is transmitted intact through cell division; defects in checkpoints lead to human disease such as cancer. Two major checkpoint pathways that have been extensively studied are the DNA damage response and the spindle assembly checkpoint. As their names imply, they have been thought to monitor distinct chromosomal events during the cell cycle. Here, we used *C*. *elegans* proliferating germ cells and human cells to investigate the role of these checkpoints when either DNA is damaged or the spindle is perturbed. We discovered that these checkpoints function together in response to these different perturbations to ensure genome integrity. Our studies have important implications for cancer treatments, as many cancer chemotherapies target one of these checkpoint pathways without consideration for the effect on the other pathway.

## Introduction

Genome integrity is monitored throughout the cell cycle by surveillance mechanisms that ensure the proper order and fidelity of DNA replication and segregation through mitosis and meiosis. This is largely achieved by the actions of two checkpoint pathways: the DNA damage response (DDR) and the spindle assembly checkpoint (SAC). As its name implies, the canonical role of the DDR is to recognize DNA damage and either arrest the cell cycle and initiate DNA repair, or induce apoptosis. The DDR is composed of a large number of proteins, prominent among them are the highly conserved protein kinases, ATM, ATR, and CHK1 [1]. An extensive body of work on these master checkpoint regulators has lead to a detailed understanding of the DDR network and its importance in monitoring and repairing DNA damage [1].

Where the DDR responds to DNA damage in several cell cycle stages, the SAC functions in metaphase to prevent premature separation of sister chromatids through inhibition of the Anaphase Promoting Complex (APC) activator CDC20 until proper chromosome alignment has been achieved [2]. It is composed of several members that are conserved from yeast to mammals: MAD1, MAD2, MAD3 (BUBR1 in mammals), BUB1 and BUB3. The intricate interactions between SAC proteins, the kinetochore and CDC20 have been studied in depth in response to metaphase microtubule disruptions [3]. Like DDR members, SAC components have garnered significant attention as they are critical for genome integrity and SAC mis-regulation has been documented in several cancers [4,5].

Although components of these extensively characterized pathways indisputably respond to the types of damage for which they are named, there is increasing evidence that the two pathways are not as distinct as previously presumed. Several DDR components (ATR, RAD9, BRCA1, ATM) are important for metaphase delay after microtubule disruptions in yeast and mammalian cells [6–9]. Moreover, DNA damage can result in metaphase arrest that is either dependent on the SAC alone [10–14] or on both the SAC and DDR [15–17]. Additionally, high throughput screens in yeast and mammalian cells identified hundreds of potential ATM/ATR target proteins including SAC or spindle-associated components (MAD1, BUB1, CENPF, CLASP 1&2, NUMA, NUSAP1) [18,19]. Thus, it appears that the DDR and SAC function together to facilitate genome integrity; however, it remains unclear the extent to which these pathways intersect at different stages of the cell cycle.

Here, we take advantage of the exceptional cytology of the *C*. *elegans* germ line, high-resolution microscopy, available mutants and the ease of RNAi to study the roles of, and interactions between, these conserved checkpoints in response to both spindle perturbations and DNA damage. Our studies reveal that DDR and SAC components together are responsible for DNA repair, chromosomal stability after metaphase disruptions and cell cycle delays in proliferating germ cells. We find that metaphase delays after spindle disruptions and stability of arrested metaphase plates are dependent on both DDR and SAC components. Additionally, the SAC component MAD-2 localizes to the nucleus in a DDR-dependent manner after DNA damage and is required for RAD-51 processing and progeny viability following DNA damage, independent of CDC20 inhibition. Interestingly, we find that histone variant CENPA is enriched at the nuclear periphery after DNA damage in a SAC- and DDR-dependent manner and CENPA is required for localization of RAD-51 to the periphery and efficient RAD-51 processing. We also provide evidence that the role of SAC in response to DNA damage is conserved in human cells. Together, we propose that DDR and SAC components interact at the kinetochore after metaphase disruptions and at the nuclear periphery after DNA damage to ensure that chromosomes are transmitted intact through the cell cycle.

## Results

### MAD-1 and MAD-2 localize along chromatin in response to lack of spindle attachments/tension and under persistent metaphase arrest once bipolar spindles have been assembled

**Fig 1:**
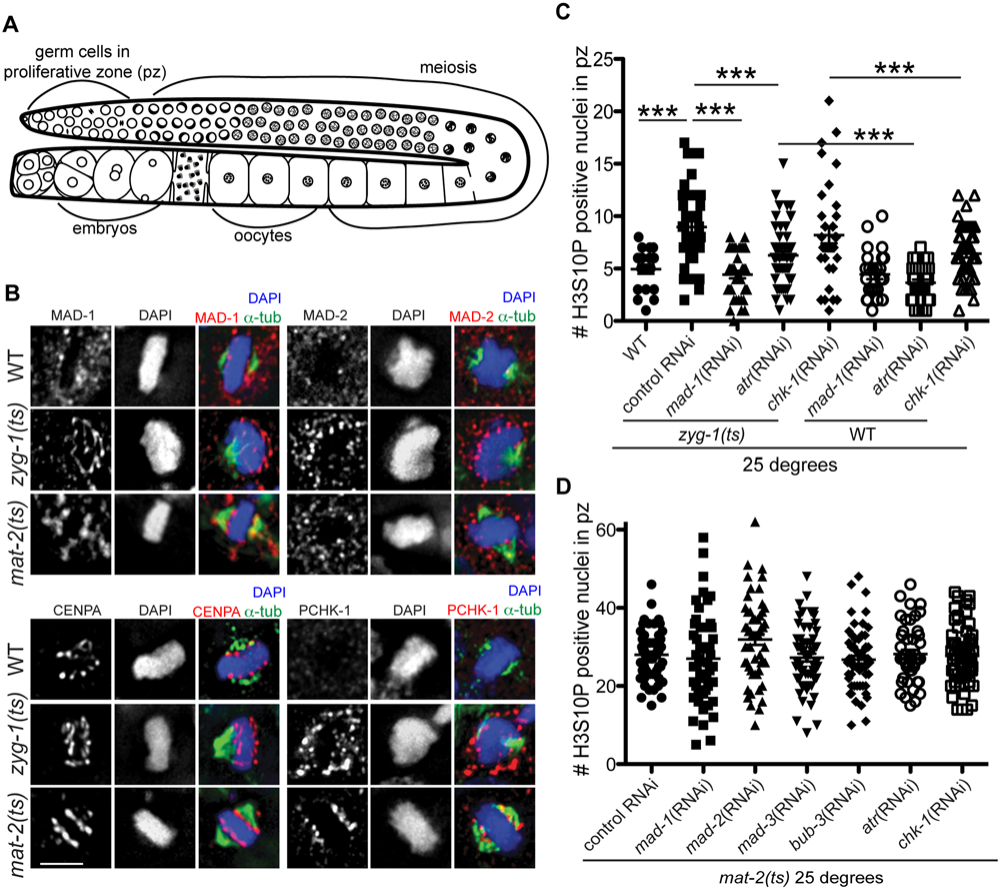
Both DDR and SAC components are responsive to metaphase perturbation. (A) Cartoon of *C*. *elegans* germ line (B) Wild-type, *mat-2(ts)*, and *zyg-1(ts)* germ lines stained with MAD-1, MAD-2, CENPA or P-CHK-1(Ser344) (red), α-tubulin (green), and counterstained with DAPI (blue) following growth at 25°. Scale bars = 5μM. (C) Quantification of H3S10P-positive nuclei per germ line in wild-type and *zyg-1(ts)* worms treated with *atr*, *chk-1*, *mad-1* or control (L4440) RNAi at 25° (n ≥ 20). *zyg-1(ts)* mean = 9.0 ±0.5 SEM vs. WT = 5.0 ±0.4, *zyg-1(ts)*; *mad-1*(RNAi) = 4.4 ±0.3, *zyg-1(ts)*; *atl-1*(RNAi) = 6.2 ±0.4, *zyg-1(ts)*; *chk-1*(RNAi) = 9.1±0.5 p = 0.88; ***p<0.0001. (D) Quantification of H3S10P positive nuclei per germ line in *mat-2(ts)* worms grown at 25° treated with control, *mad-1*, *mad-2*, *mad-3*, *bub-3*, *atr*, or *chk-1* RNAi (n≥48). H3S10P counts between *mat-2(ts)* and RNAi depletions were not significant except for *mad-2*(RNAi), which had more H3S10P than control RNAi, p = 0.02, indicating efficient arrest.

To analyze the *in vivo* roles of the SAC and DDR, we examined proliferating cells in the *C*. *elegans* germ line, which is arranged in a spatiotemporal pattern (Fig 1A) and is amenable to genetic and cytological analyses. Further, this is the only tissue in the adult worm that is actively dividing. We first examined the localization of SAC components MAD-1 and MAD-2 (also known as MDF-1 and MDF-2) after metaphase perturbations. To that end, we disrupted metaphase using two different conditional alleles: *zyg-1(b1)*[referred to as *zyg-1(ts)*[20,21]] and *mat-2(ax102)*[referred to as *mat-2(ts)*[22]] as microtubule-inhibiting drugs, which have traditionally been used to induce SAC activation, prevent dynamics of the mitotic spindle and have potential off-target effects. ZYG-1 is functionally related to PLK4 and is required for centrosome duplication [21]. Inactivation of ZYG-1 leads to monopolar spindles, loss of proper spindle attachment/tension and a SAC-dependent metaphase delay [23,24]. On the other hand, MAT-2 is a component of the APC, a E3 ubiquitin ligase responsible for removal of sister chromatid cohesion at the metaphase to anaphase transition, and its inactivation presumably arrests metaphase progression downstream of microtubule attachment and achievement of tension [25].

Using antibodies directed against MAD-1 [26] and MAD-2 [27] we observed a modest enrichment of both of these SAC components along the face of chromatin not associated with the monopolar spindle (i.e., lacking attachment/tension) in *zyg-1(ts)* [23,27] (Fig 1B). The staining pattern of MAD-1 and MAD-2 in proliferating germ cells was consistent with holocentric kinetochore localization, as a similar pattern was observed for centromere-specific histone CENPA (HCP-3 in *C*. *elegans*)[28–30](Fig 1B). Although available antibodies precluded co-staining CENPA and MAD-1 (or MAD-2) with two different secondary antibodies to distinguish the signal, we co-stained with the same secondary antibody to determine whether there was a difference in the staining pattern, which would suggest distinct localization. We saw no significant difference in the extent of staining of CENPA compared to MAD-1/CENPA (S1A Fig), consistent with MAD-1/2 enrichment at the kinetochore (marked by CENPA) in proliferative zone germ cell nuclei. Further, although MAD-1/2 was enriched along the chromatin opposite the spindle (i.e., lacking tension), kinetochores were present on both faces of the chromatin in *zyg-1(ts)* as revealed by staining with CENPA (Fig 1B) and the outer kinetochore component, NDC-80 (S1B Fig), suggesting that MAD-1/2 is enriched on kinetochores lacking tension or microtubule attachment.

MAD-2 localization has been characterized in *C*. *elegans* embryos expressing transgenic GFP::MAD-2. We noted that the accumulation of endogenous MAD-2 to chromatin that we observed in the germ line was not as robust as reported in GFP::MAD-2 embryos [27]. To determine whether the extent of accumulation represented a difference in checkpoint signaling in the germ line or was a result of GFP::MAD-2 overexpression, we compared endogenous embryonic MAD-1/2 localization and localization in embryos expressing GFP::MAD-2. To that end, we induced a SAC-dependent metaphase arrest in early embryos by depleting CyclinB3 (CYB-3 in *C*. *elegans*), which results in arrest prior to the four-cell stage due to improper formation of the kinetochore [31], and observed a similar pattern of staining in embryos lacking proper spindle attachments/tension (S1C Fig) as we did in the germ line. Further, GFP::MAD-2 embryos showed more robust staining upon activation and GFP::MAD-2 was observed on chromatin even in the absence of spindle perturbation in the one-cell embryo, suggesting that the difference in accumulation is due to overexpression of GFP::MAD-2 and not an inherent difference between embryonic and germline SAC signaling (S1C Fig; [26,31]).

We also examined the localization of MAD-1/2 upon inactivation of MAT-2/APC, which results in stable metaphase arrest. We observed enrichment of MAD-1, and to a lesser extent MAD-2, to the lengths of chromatin following inactivation of MAT-2/APC, suggesting SAC is also activated in response to persistent metaphase arrest after chromosomes have bi-oriented (Fig 1B). Together, these results suggest that under both tension/attachment defects and metaphase arrest, MAD-1 and MAD-2 are enriched on the kinetochore.

### Activated CHK-1 also shows kinetochore-like localization in response to lack of spindle attachments/tension and under persistent metaphase arrest once chromosomes have bi-oriented

We next examined whether the DDR, like the SAC, responded to mitotic spindle defects. In response to DNA damage, ATR (ATL-1 in *C*. *elegans*) phosphorylates CHK-1 at Ser345 (Ser344 in *C*. *elegans*) and activates a signaling cascade to arrest the cell cycle and activate DNA repair pathways [32]. To determine whether DDR components are activated and localized to kinetochores following ZYG-1 or MAT-2/APC inactivation, we used an antibody against human P-CHK-1(Ser345) [33,34]. After ZYG-1 inactivation, we observed P-CHK-1(Ser344) on the outward faces of chromosomes, in a pattern similar to MAD-1/2 and consistent with kinetochore localization (Fig 1B). CHK-1 phosphorylation was specific to monopolar spindles as we did not observe P-CHK-1(Ser-344) on chromatin in wild-type metaphase cells (Fig 1B), suggesting that the DDR is activated in response to monopolar spindles. We also observed localization of P-CHK-1(Ser344) to chromatin following MAT-2/APC inactivation in a pattern similar to MAD-1 (Fig 1B), suggesting the DDR is also activated in response to persistent metaphase arrest once tension has been achieved.

### SAC and DDR components mediate metaphase delay in response to lack of spindle attachments/tension

To determine whether the DDR, like the SAC, mediated a metaphase delay in response to insufficient spindle attachments, we analyzed cell cycle kinetics by monitoring the number of nuclei enriched for phosphorylation of Serine 10 on Histone H3 (H3S10P), a marker of pro-metaphase/metaphase [35]. Elevated levels of H3S10P are indicative of a metaphase delay when ZYG-1 is inactivated [24]. As expected, we observed an increase in H3S10P-positive nuclei following ZYG-1 inactivation, which was abrogated upon depletion of MAD-1 (Fig 1C), indicating a SAC-dependent metaphase delay. Consistent with DDR activation in *zyg-1(ts)* as monitored by P-CHK-1(Ser344) along the length of the chromosomes, metaphase delay was impaired following depletion of ATR (Fig 1C), although not to the extent of SAC depletion. These results suggest that ATR helps to facilitate cell cycle delay in response to defects in attachment or tension on the spindle (Fig 1C). Interestingly, depletion of CHK-1 did not abrogate delay suggesting that ATR-mediated cell cycle delay is not mediated solely through phosphorylation of CHK-1 (Fig 1C). CHK-1 was efficiently depleted as monitored by failure to induce cell cycle arrest in response to stalled replication forks (S1D Fig). Thus under these RNAi conditions, ATR but not CHK-1 is necessary for metaphase delay in response to monopolar spindles. As both SAC and DDR components are also enriched on chromatin upon inactivation of MAT-2, we investigated whether depletion of DDR or SAC affected metaphase arrest in *mat-2(ts)* worms (Fig 1D). We saw no difference in H3S10P after depletion of SAC or DDR following inactivation of MAT-2, suggesting SAC and DDR primarily affect metaphase delays associated with spindle defects but not persistent metaphase arrest of bi-oriented chromosomes.

### SAC and DDR components are required to maintain chromosome and spindle stability during persistent metaphase arrest

**Fig 2:**
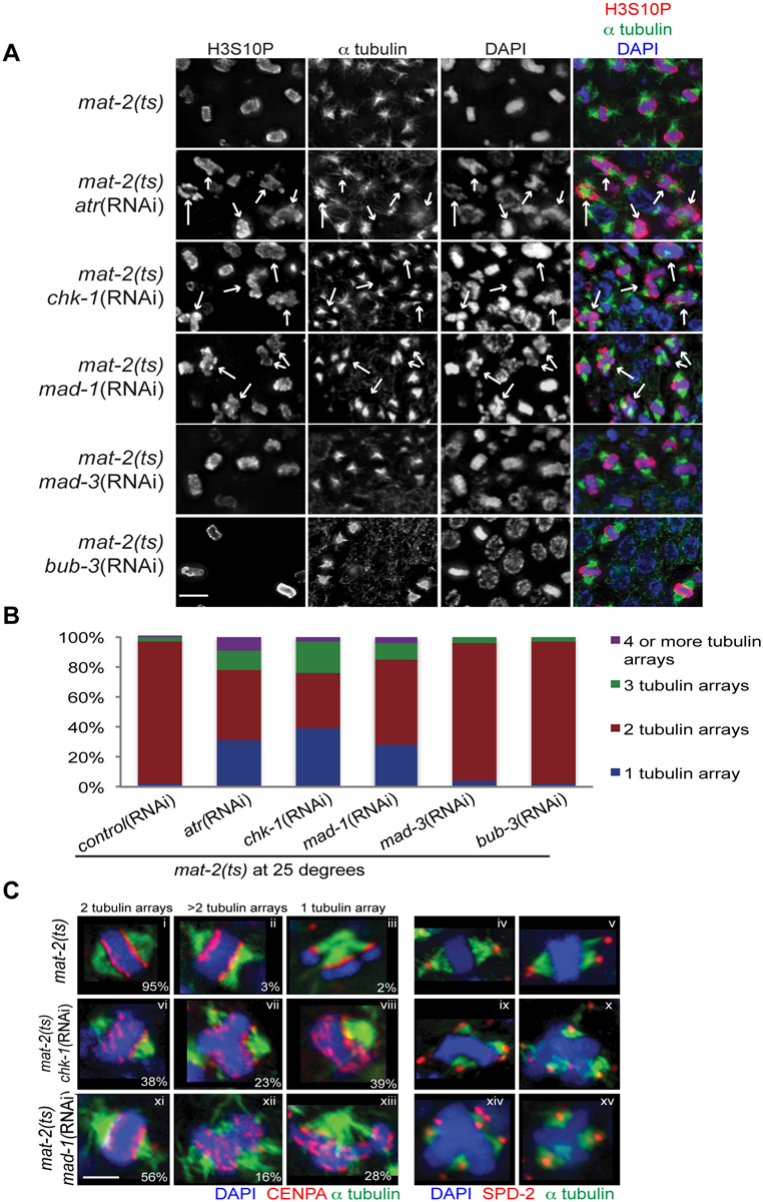
Both DDR and SAC depletion lead to aberrant spindles and DNA morphology during metaphase arrest. (A) *mat-2(ts)* germ lines treated with either control, *atr*, *chk-1*, *mad-1*, *mad-3* or *bub-3*(RNAi) at 25° and stained with H3S10P (red), α-tubulin (green) and DAPI (blue). Arrows point to nuclei with aberrant DNA morphology and multiple or singular tubulin arrays. Scale bar 5μM. (B) Percentage of tubulin arrays in proliferative zones of the above genotypes at 25° (n≥10 germ lines). Percent of 2-tubulin-arrays is significantly different between *mat-2(ts)*;control(RNAi) and *mat-2(ts);atr*(RNAi), *mat-2(ts);chk-1*(RNAi), *mat-2(ts);mad-1*(RNAi), all p<0.0001 (Fishers exact test). (C) *mat-2(ts);chk-1*(RNAi), *mat-2(ts);mad-1*(RNAi), or *mat-2(ts)*;control(RNAi) metaphase nuclei stained with CENPA or SPD-2 (red), α-tubulin (green) and DAPI (blue) at 25°. The frequency of different classes is indicated. Scale bar 2μM.

To determine whether the DDR facilitated metaphase chromosome stability during prolonged arrest, once chromosomes have achieved bi-orientation, we depleted DDR components ATR and CHK-1 individually followed by inactivation of MAT-2/APC. To assess stability of the metaphase plate we examined both chromatin morphology (H3S10P and CENPA) and spindle formation and stability (α-tubulin and SPD-2 [pericentriolar material]; [36]). Following MAT-2/APC inactivation, H3S10P-positive nuclei were predominantly condensed into tight bars aligned on the metaphase plate with organized CENPA and two tight triangular bi-oriented tubulin arrays with two SPD-2 foci (95% of metaphase nuclei; Fig 2A and 2C). In contrast, depletion of either ATR or CHK-1 during MAT-2/APC inactivation resulted in 62% and 44% of H3S10P-positive nuclei that contained decondensed chromatin (CENPA) and either one or more than two α-tubulin arrays and SPD-2 foci, respectively (p<0.0001; Fig 2A and 2C). To further analyze the severity of metaphase abnormalities, we calculated the percent of α-tubulin array classes in the different genotypes and found that depletion of the DDR during metaphase arrest significantly compromised the ability to maintain a stable metaphase plate with bi-oriented tubulin arrays (Fig 2B). This was specific to persistent metaphase arrest as neither inactivation of ATR or CHK-1 induced significant metaphase defects at the non-permissive temperature in an otherwise wild-type worm (S2A and S2B Fig).

We next analyzed the requirement for the SAC during prolonged metaphase arrest. To that end, we depleted SAC components MAD-1 or MAD-2 in *mat-2(ts)* worms and monitored H3S10P, CENPA, α-tubulin and SPD-2 to analyze chromosome and spindle morphology. As with depletion of DDR components, depletion of SAC proteins MAD-1 or MAD-2 led to metaphase plate instability and an increase in single and multiple α-tubulin arrays following MAT-2/APC inactivation (Figs 2A–2C and S2), suggesting that these SAC components are required to stabilize metaphase plates under persistent arrest.

When kinetochore-spindle attachments have not been achieved or bi-polar tension is absent, MAD-1-MAD-2 interactions at the kinetochore initiate the formation of the mitotic checkpoint complex (MCC) (MAD-2, MAD-3, BUB-3) in the nucleoplasm to inhibit APC activity and delay anaphase [37]. As MAT-2/APC activity is downstream of canonical SAC activation, we hypothesized MAD-1 and MAD-2 function in a novel pathway to ensure metaphase stability independent of the MCC. To test this, we depleted MAD-3 or BUB-3 in *mat-2(ts)* worms and examined H3S10P, CENPA, α-tubulin and SPD-2. In contrast to what was observed upon inactivation of MAD-1 or MAD-2, chromosome morphology and α-tubulin arrays appeared similar to wild type following MAD-3 and BUB-3 depletion in *mat-2(ts)*(Fig 2A and 2B). To determine SAC RNAi efficiency, we assayed embryonic cell division after depleting CyclinB3, which induces a SAC-dependent metaphase arrest [31]. Co-depletion of CyclinB3 with all SAC components resulted in a similar failure to induce metaphase arrest (S2C Fig), indicating efficient knockdown. These data suggest that MAD-1 and MAD-2, but not other members of the MCC, play a novel role in maintaining metaphase plate stability once microtubule attachment/tension has been achieved. Taken together, these results indicate that SAC and DDR components both mediate chromosome stability throughout metaphase.

### MAD-2 is enriched at the nuclear periphery in response to DNA damage

**Fig 3:**
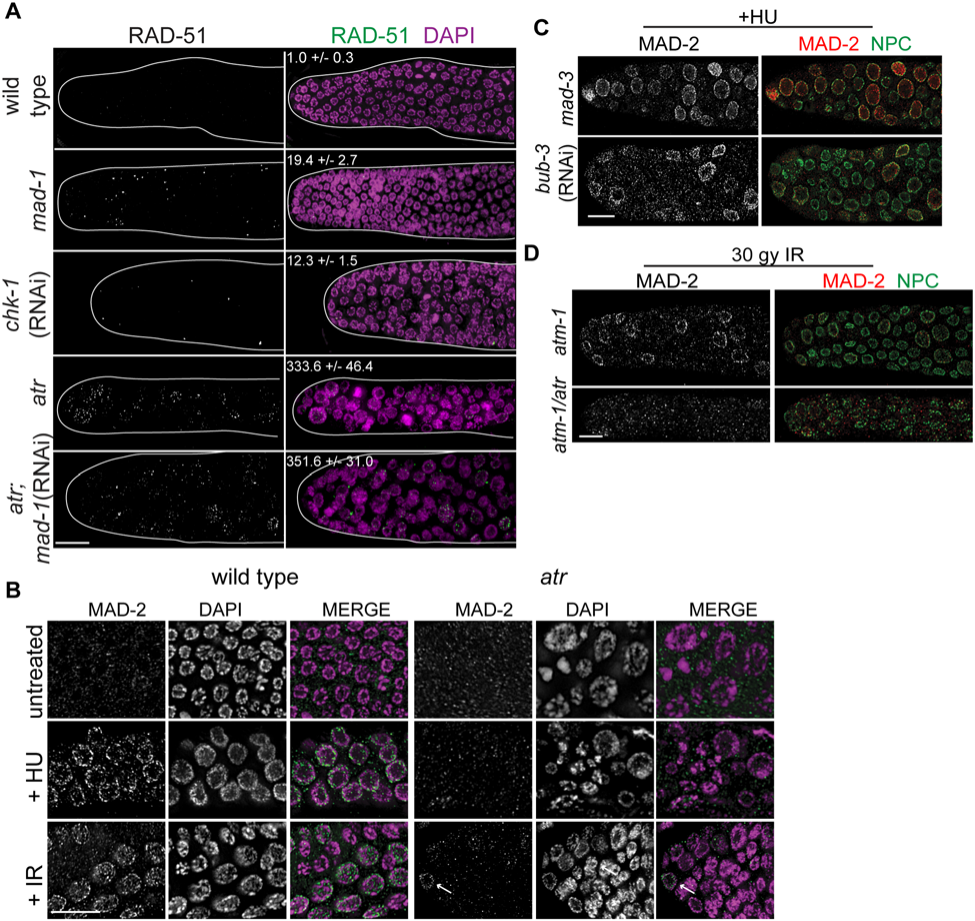
MAD-2 is enriched at the nuclear periphery after DNA damage in an ATR- dependent manner. (A) Wild-type, *mad-1(gk2)*, *chk-1*(RNAi), *atr(tm853)*, and *atr(tm853);mad-1*(RNAi) germ lines stained with RAD-51 (green) and counterstained with DAPI (magenta). Numbers to right indicate mean RAD-51 foci per germ line ±SEM (n = 10). (B) MAD-2 (green) staining in wild-type or *atr(tm853)* germ lines in the absence of damage or after HU or IR treatment counterstained with DAPI (magenta). Arrow indicates cell with nuclear MAD-2 staining. (C) Germ lines of *mad-3(ok1580)* and *bub-3*(RNAi) worms after HU stained with MAD-2 (red) and NPC (green). (D) MAD-2 localization after IR is ATR and ATM dependent. Although some MAD-2 (red) can still localize to the nuclear periphery (NPC, green) in *atm-1(gk186)* after 30 gy of IR, MAD-2 localization is abolished in the *atm-1(gk186); atr(tm853)* double mutant. Scale bars = 10μm.

Our results indicate that the DDR and SAC function together throughout metaphase to ensure chromosome stability. To explore the possibility that SAC functions outside of metaphase in response to DNA damage similarly to the DDR, we monitored spontaneous DNA damage in proliferating germ cells by examining the appearance of RAD-51 recombinase, which marks regions of single-stranded DNA induced by stalled replication forks or double strand breaks (DSBs). As expected, germ lines depleted for DDR components CHK-1 or ATR had significantly elevated levels of RAD-51 compared to wild type (p<0.0001; Fig 3A). *mad-1* mutants also had significantly elevated levels of RAD-51 (p<0.0001; Fig 3A), suggesting that the SAC plays a role in DNA damage signaling and/or repair. *atr* mutants and *atr;mad-1*(RNAi) double mutants had similar levels of spontaneous RAD-51 foci, suggesting ATR and MAD-1 could be functioning in the same pathway to monitor spontaneous DNA damage.

We next examined whether SAC components function with the DDR in response to induced DNA damage. To that end, we monitored localization of SAC components MAD-2 and MAD-1 upon induction of replication fork stalling/collapse by treating worms with the ribonucleotide reductase inhibitor, hydroxyurea (HU), which results in an S-phase arrest and enlarged nuclei [38], or after exposure to ionizing radiation (IR), which induces DSBs and leads to a G2 arrest [39]. In wild-type worms, MAD-2 was observed in a punctate pattern throughout the cytoplasm (Fig 3B). Following treatment with HU (25mM) or IR (30 Gy), MAD-2 was enriched at the nuclear periphery, as was the majority of genomic DNA (Fig 3B); subsequent analyses suggested that this reflects association with the nuclear periphery (see below). MAD-2 accumulated at the nuclear periphery in response to DNA damage and not cell cycle alteration, as depletion of Cyclin E or cell cycle dependent kinase CDK-2 did not result in MAD-2 accumulation at the nuclear periphery (S3A Fig), although the cell cycle was perturbed as monitored by H3S10P (wild type = 5.0±0.5, *cye-1*(RNAi) = 2.9 ±0.7, p = 0.02; *cdk-2*(RNAi) = 1.7±0.6, p<0.0001).

In interphase, MAD-1 is tethered to the nuclear periphery by the nuclear pore component NUP-107 (NPP-5 in *C*. *elegans*) [40] and it remains enriched at the nuclear periphery following treatment with either HU or IR (S3 Fig). However, in the absence of NUP-107, neither MAD-1 nor MAD-2 were enriched at the nuclear periphery (S3B Fig), suggesting that MAD-1 is required to tether MAD-2 to the nuclear periphery following DNA damage. On the other hand, the MCC components MAD-3 and BUB-3 were not required for MAD-2 localization to the nuclear periphery after HU (Fig 3C).

As MAD-1 normally resides at the nuclear periphery in interphase yet only interacts with MAD-2 at the nuclear periphery following DNA damage, we explored the possibility that the nuclear enrichment of MAD-2 was dependent on the DDR. Indeed, while MAD-1 was still tethered at the nuclear periphery (S3C Fig), MAD-2 was not enriched at the nuclear periphery following HU treatment in the absence of ATR (Fig 3B). After IR, MAD-2 was not enriched at the nuclear periphery in the majority of proliferating germ cell nuclei in the absence of ATR; however, in a few proliferating germ cell nuclei MAD-2 enrichment was observed. We hypothesized this was due to the activity of ATM, the related and partially redundant DDR kinase that responds primarily to DSBs [1]. Consistent with this, MAD-2 nuclear localization was completely abolished in the ATR/ATM double mutant after IR (Fig 3D). These results indicate that MAD-2 becomes enriched at the nuclear periphery in response to DNA damage in a DDR-dependent manner.

### SAC-mediated CDC20 inhibition is required to delay M phase when replication is perturbed

**Fig 4:**
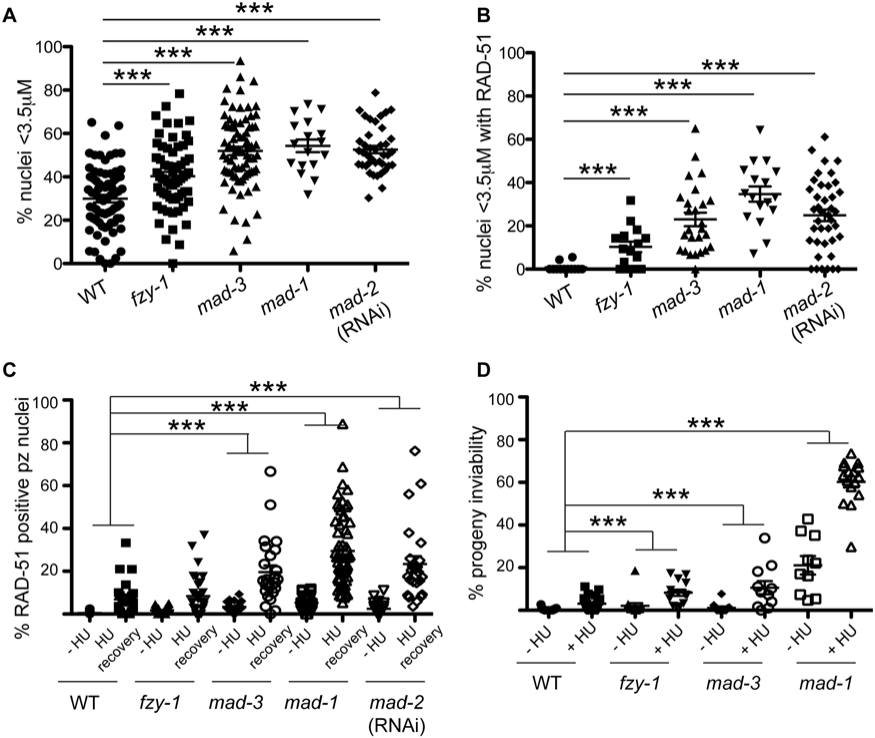
SAC components function in part by delaying metaphase in the presence of DNA damage. (A) Percent of nuclei smaller than 3.5μM, the average diameter of nuclei in untreated germ lines, after release from HU in wild-type, *fzy-1(av15)*, *mad-3(ok1580)*, *mad-1(gk2)*, and *mad-2*(RNAi) germ lines: wild type = 30.0±1.7%, *fzy-1(av15)* = 40.2±2.0%; *mad-3* = 52.0±1.9%; *mad-1* = 54.3±2.9%; *mad-2* = 52.6±1.7% (n≥24). (B) Percent of nuclei that are smaller than 3.5μM that have at least 1 RAD-51 focus in wild-type, *fzy-1(av15)*, *mad-3(ok1580)*, *mad-1(gk2)*, and *mad-2*(RNAi) germ lines: wild type = 0.8±0.6%; *mad-3* = 23.0±3.1%; *mad-1* = 34.7±3.5%; *mad-*2 = 24.9±2.6% (n≥17). (C) Percent of nuclei that have at least 1 RAD-51 focus either—HU or 24hrs recovery in wild-type, *fzy-1(av15)*, *mad-3(ok1580)*, *mad-1(gk2)*,and *mad-2*(RNAi) germ lines: wild type:-HU = 0.4±0.1% vs. +HU 6.9±1.7%, Δ6.5%; *fzy-1(av15)*:-HU = 1.7±0.3% vs. +HU = 8.4±1.8%, Δ6.7%; *mad-3*:-HU = 3.4±0.5% vs. +HU = 19.7±3.1%, Δ16.3%; *mad-1*:*—*HU = 5.3±0.5% vs. + HU = 29.5±2.3%, Δ24.2%; *mad-2*:—HU = 2.5±0.5% vs +HU±3.7%±23.4, Δ20.9%; (n≥ 15). (D) Percent progeny inviability—HU and after HU exposure in wild type, *fzy-1(av15)*, *mad-3(ok1580)*, and *mad-1(gk2)*; wild type:-HU = 0.4±0.1 vs. +HU = 3.1±0.6%, Δ2.7%; fzy-1(av15):-HU = 2.1±1.3% vs. +HU = 8.3/-1.4%, Δ6.2%; *mad-3*:*—*HU = 1.2±0.6% vs. +HU = 10.6±3.1%, Δ9.4%, p = 0.005; *mad-1*:—HU = 21.1±4.3% vs. +HU = 60.2±2.6% Δ39.1% (n≥10). We did not include *mad-2*(RNAi) due to high levels of sterility and low brood sizes. ***p<0.0001 (two-way ANOVA). Error bars indicate SEM.

Recent studies in yeast and mammalian cells have shown that the SAC is active in interphase to inhibit the onset of mitosis [16,41]. To determine whether the enrichment of MAD-2 at the nuclear periphery reflects anticipation of a need to delay mitosis until replication and repair have been completed, we monitored cell cycle progression following removal of HU in wild type and *sac* mutants. All genotypes examined were competent for cell cycle arrest following HU as monitored by nuclear morphology and number of H3S10P positive nuclei (wild type = 0.2±0.09, *mad-3* = 0.2±0.1, *mad-1* = 0.1±0.05). Following release from HU, we found accelerated mitosis in *sac* mutants as reflected in both the appearance of small nuclei (<3.5μm), indicative of recently divided cells (Fig 4A), and a decrease in H3S10P-positive cells compared to wild type, indicating a greater rate of mitosis completion (e.g., wild type = 4.9±0.4; *mad-3* = 3.3±0.3; p = 0.0054). Further, elevated levels of RAD-51 were observed in the small nuclei of *sac* mutants, suggesting that HU-induced stalled/collapsed forks had not been repaired in recently divided cells (Fig 4B). We also examined the persistence of RAD-51 foci in proliferating germ cells and progeny viability following release from HU. Consistent with a failure to delay mitosis, *sac* mutants had both elevated RAD-51 foci (Fig 4C) and increased progeny inviability following release from HU compared to wild type (Fig 4D).

When the SAC is activated in metaphase, the MCC complex inhibits the APC activator, CDC20, to prevent the metaphase to anaphase transition. To determine whether the defects observed in *sac* mutants were the consequence of a failure to inhibit CDC20 to block APC activity, we monitored cell cycle kinetics and RAD-51 foci in worms harboring a CDC20 mutation [*fzy-1(av15)*], which renders the worm incompetent for metaphase delay (S2C Fig) [42]. We found that similar to *sac* mutants, *fzy-1(av15)* mutants were competent for arrest with HU (H3S10P = 0.1±0.04) yet had accelerated mitosis following release from arrest as monitored by small (Fig 4A), and H3S10P-positive nuclei (2.3±0.3 vs. wild type = 4.9±0.4, p<0.0001). Additionally, the recently divided nuclei had elevated levels of RAD-51 (Fig 4B), and progeny viability was reduced in the absence of *fzy-1(av15)* following release from HU (Fig 4C and 4D), although not to the extent observed in *sac* mutants. Interestingly, after extended HU recovery, *fzy-1(av15)* worms were largely able to repair the HU-induced damage, as RAD-51 levels were equivalent to wild type (Fig 4C). These results suggest that the SAC functions in interphase in part to prevent mitosis in the presence of incompletely replicated or damaged DNA.

### SAC components promote DNA repair independent of CDC20 inhibition

**Fig 5:**
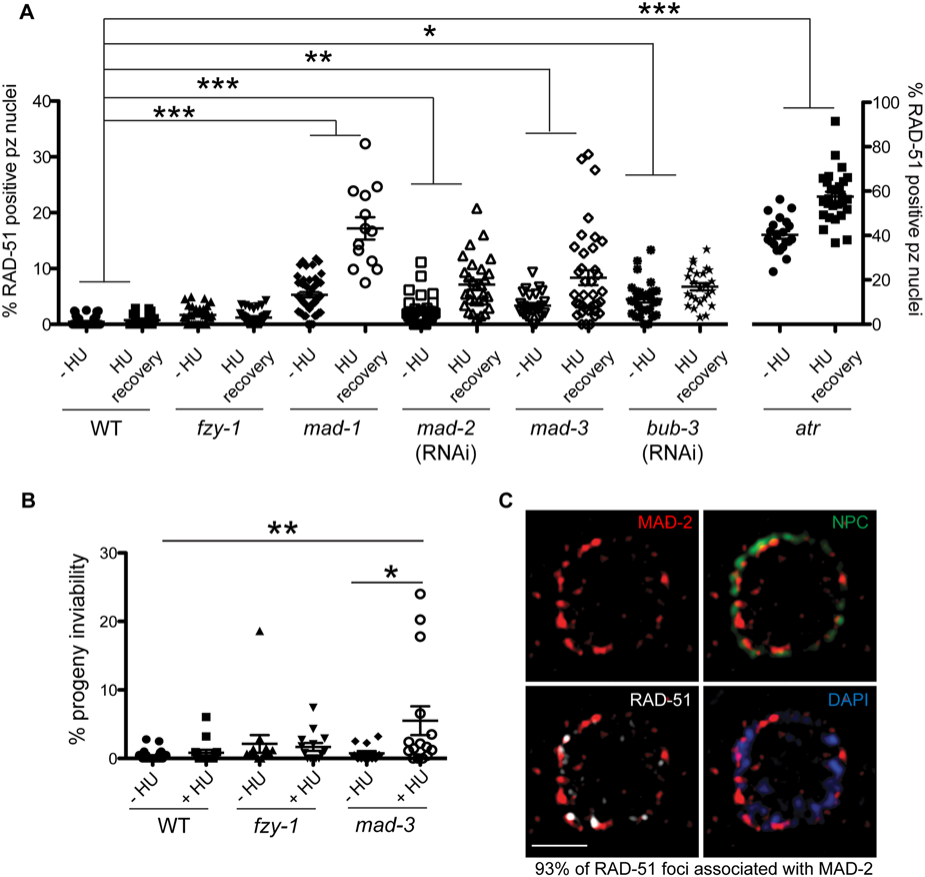
Both DDR and SAC components are required for efficient DNA damage repair and progeny viability after HU. (A) Percent of nuclei that contain at least 1 RAD-51 focus—HU or 16 hours after 5mM HU recovery in wild-type, *fzy-1(av15)*, *mad-1(gk2)*, *mad-2*(RNAi), *mad-3(ok1580)*, *bub-3*(RNAi), *and atr(tm853)* worms. Difference between—HU and +HU is statistically different in all genotypes except wild type and *fzy-1(av15)*, p< 0.02 (two-way ANOVA)(n≥13). (B) Percent progeny inviability—HU and after 5mM HU exposure in WT *and mad-3(ok1580)*; n≥13. *p<0.05, **p<0.001, ***p<0.0001 (two-way ANOVA). Error bars represent SEM. (C) Proliferative zone nucleus of WT worm stained for RAD-51 (white), NPC (red), MAD-2 (green) and DAPI (blue) in the presence of HU. Image represents one slice of a z-stack taken on SIM. Scale bar = 5μm.

During replication stress, stalled forks must be stabilized to facilitate fork restart during recovery. Failure in fork stabilization or restart leads to DNA breaks [43], which results in elevated RAD-51 foci. We observed many more RAD-51 foci in *sac* mutants compared to *fzy-1(av15)* (Fig 4C), suggesting that SAC has additional roles in DNA repair independent of mitotic delay. To investigate this we treated worms with a 2 hour pulse of 5mM HU and monitored RAD-51 foci appearance and disappearance and progeny viability upon release from HU. This dose had no effect on wild-type worms with respect to either cell cycle kinetics (H3S10P after 6hr recovery = 5.6±0.3 vs.—HU = 5.0± 0.3, p = 0.12) or progeny viability (Fig 5A and 5B). Analysis of RAD-51 revealed that approximately 17% of wild-type proliferating germ cells have RAD-51 immediately following release from HU, this peaks to 21% after 2 hours and then declines to almost basal levels by 6 hours after HU exposure (2%), and by 16 hours only 0.7% of cells have RAD-51 foci (Figs 5A and S4A). In *mad-1* mutants the levels of RAD-51 foci were initially lower (9%) than in wild type after HU but then gradually increased throughout the time course (17% at 16 hours) (Figs 5A and S4A). The pattern of RAD-51 increasing over time in *mad-1* mutants was very similar to the ATR mutant although we observed an overall higher basal level of RAD-51 foci in the absence of ATR (Figs 5A and S4A). After 16 hours of HU recovery, all the *sac* mutants investigated (*mad-1*, *mad-2*(RNAi), *mad-3*, *bub-3*(RNAi) had persistent RAD-51 foci (Fig 5A), suggesting that similar to the DDR, SAC promotes fork stabilization/restart.

In contrast to *sac* mutants, *fzy-1(av15)* was competent for repair as monitored by RAD-51 foci during low dose HU (Figs 5A and S4A), suggesting that SAC components do not function by inhibiting CDC20 in response to modest levels of DNA damage as they do during spindle perturbations or in the presence of excessive DNA damage. Further, unlike *sac* mutants, we observed no effect on progeny viability following low dose exposure to HU in *fzy-1(av15)* mutants (-HU = 2.1±1.3% vs. +HU = 1.7±0.6%, p = 0.76), suggesting that SAC does not function to inhibit CDC20 when modest levels of DNA damage are induced. Defects in DNA damage repair were not limited to replication stresses in SAC mutants, as RAD-51 foci also persisted in germ cell nuclei after recovery from 30gy of IR (S4E Fig).

As DNA damage sensor and signal transducers ATR and ATM are required for MAD-2 relocalization to the nuclear periphery, and given the role for SAC components in DNA repair, we hypothesized that MAD-2 foci colocalize with sites of DNA damage. To test this hypothesis, we used high-resolution structured illumination microscopy (SIM) to determine the localization of MAD-2 and RAD-51 relative to the nuclear periphery as marked by nuclear pores (NPC). We found that approximately 90% of MAD-2 was at the nuclear periphery, juxtaposed to nuclear pore complexes (Fig 5C). RAD-51 foci were also enriched at the nuclear periphery, and 93% of RAD-51 foci were associated with a patch of MAD-2 (Fig 5C). These results suggest that MAD-2 interacts with damaged DNA in a DDR-dependent manner, to aid in downstream cellular responses to damage.

### The histone variant CENPA facilitates DNA repair following damage and is enriched at the nuclear periphery in a SAC- and DDR-dependent manner

**Fig 6:**
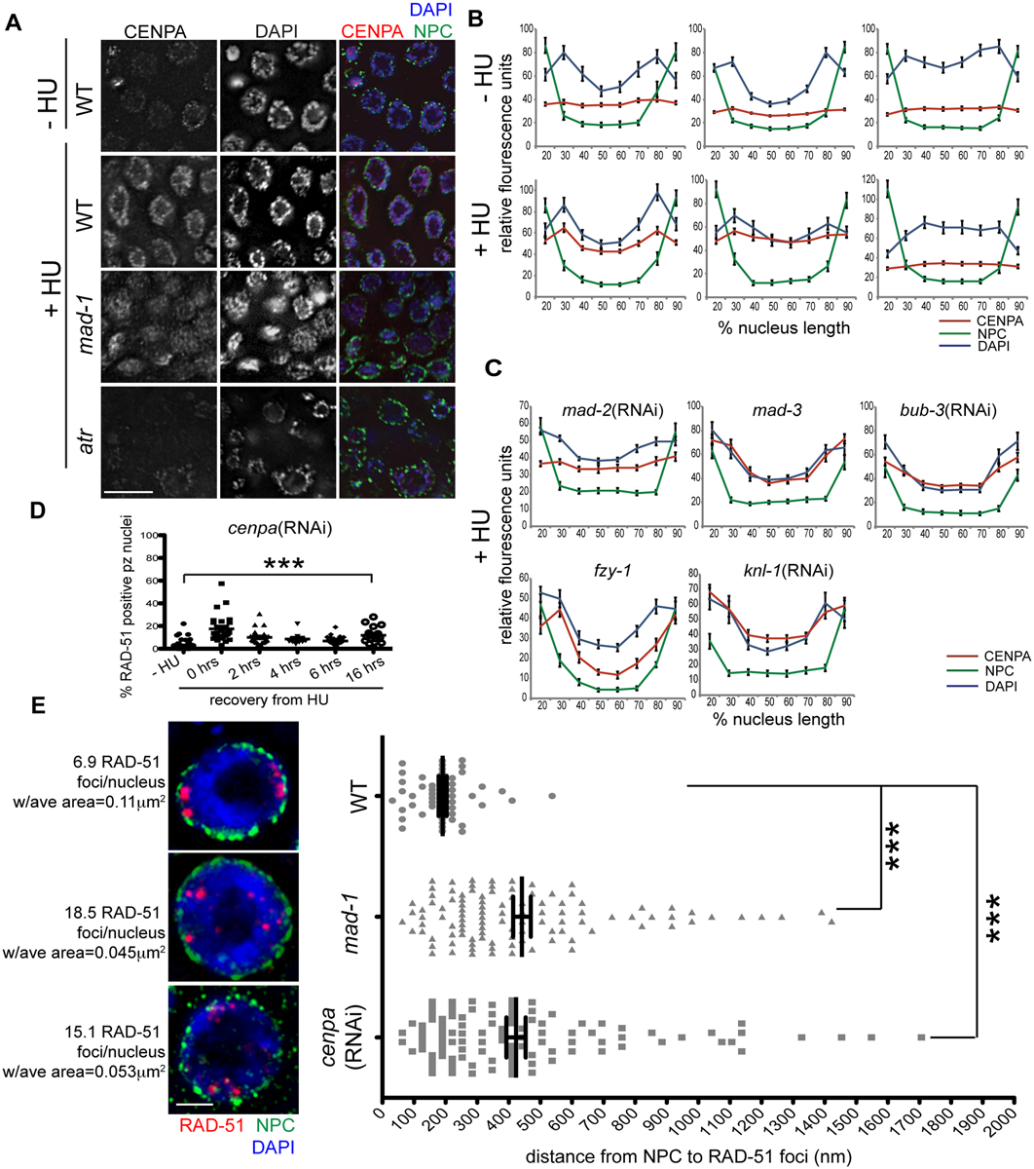
CENPA is regulated by both DDR and SAC components and is required for recruitment of RAD-51 to the nuclear periphery and efficient DNA damage repair. (A) Proliferative zones of wild-type, *mad-1(gk2)* and *atr(tm853)* worms treated with HU and stained for CENPA (red), Mab414 (NPC) (green) and DAPI (blue). (B) Quantification of the average fluorescence intensity of CENPA, MAb414, and DAPI across the nucleus length binned in 10% increments-HU for wild type, *mad-1(gk2)*and *atr(tm853)*, and +HU for the above genotypes (n≥30). (C) Quantification of the average fluorescence intensity of CENPA and Mab414 across the nucleus length binned in 10% increments for *mad-2*(RNAi), *mad-3(ok1580)*, *bub-*3(RNAi), *knl-1*(RNAi) and *fzy-1(av15)* in the presence of HU (n≥30). (D) Percent of nuclei that contain at least 1 RAD-51 focus—HU or 0, 2, 4, 6, or 16 hours after release from 5mM HU with *cenpa*(RNAi); n≥10. Scale bars 10μm. (E) SIM images of a single nucleus from wild-type, *mad-1(gk2)* and *cenpa*(RNAi) worms treated with HU. Next to each image is the average number of RAD-51 foci observed in each genotype, as well as the average area of each RAD-51 focus (n = 4 germ lines). Images represent a projection of 3 z sections. Graph indicates the distance in nanometers between NPC and RAD-51 foci in wild-type, *mad-1(gk2)* and *cenpa*(RNAi) worms after HU. Scale bar 2 μm. Error bars indicate SEM. ***p<0.0001

To determine the mechanism by which the SAC and DDR cooperate to repair damage at the nuclear periphery, we examined the localization of the histone variant CENPA, as it associates with both DNA and SAC during metaphase. Further, CENPA has been shown to localize to sites of DNA damage in mammalian cells [44,45]. Following exposure to HU or IR, CENPA was enriched at the nuclear periphery, along with bulk DNA, in the majority of proliferative zone nuclei (Figs 6A, 6B and S5A). This was most likely the result of redistribution of the CENPA pool, as the overall steady state level of CENPA was not altered in the presence of DNA damage (S5B Fig). In the absence of either MAD-1 or MAD-2, CENPA was redistributed to the nucleus but no longer enriched at the nuclear periphery after HU; the DAPI signal was partially enriched at the periphery in these mutants but not to the extent of wild type (Fig 6A–6D). When ATR function was abrogated, CENPA, along with bulk DNA, were neither redistributed nor tethered to the nuclear periphery in response to HU (Figs 6A, 6B and S5E). In contrast, loss of MCC components MAD-3 and BUB-3 did not alter CENPA localization to the periphery after HU (Fig 6C). Additionally, CENPA enrichment at the nuclear periphery was not altered in *fzy-1(av15)*, indicating that SAC-dependent CENPA localization is not mediated through CDC20 inhibition (Fig 6D).

The kinetochore is a large multilayered complex; CENPA makes up the centromeric core on which the rest of the structure is built [28]. To determine whether CENPA redistribution following DNA damage serves to nucleate the rest of the kinetochore or represents a novel function in DNA repair, we examined the localization of the outer kinetochore component NDC-80 before and after HU treatment. We found that there was no enrichment of NDC-80 on chromatin following exposure to HU (S5C Fig), suggesting CENPA and MAD-1/MAD-2 are interacting through novel DNA-damage-induced components, independent of canonical kinetochore components. Consistent with this, partial depletion of CENPA, but not KNL-1, which is essential for the kinetochore assembly pathway downstream of CENPA [2,46], leads to a defect in DNA repair after HU (% of RAD-51 positive nuclei- *knl-1*:-HU = 1.5±0.4% vs. +HU = 1.3±0.4%, p = 0.72; Figs 6E and S5D). Further, inactivation of KNL-1 did not alter CENPA enrichment at the nuclear periphery in response to DNA damage (Fig 6C and 6D).

The enrichment of CENPA at the periphery could be an indirect consequence of how DNA is configured within the nucleus in the presence of HU (Fig 6B). Alternatively, CENPA could be associated specifically with damaged DNA, which is enriched at the periphery. To distinguish between these possibilities, we treated wild type, *mad-1* mutants and *cenpa*(RNAi) depleted animals with HU and measured the distance between the nuclear periphery and RAD-51 foci in proliferating germ cells using high resolution SIM. We found that RAD-51 foci were on average 190.8±13 nm away from nuclear pores in WT compared to 441.8±28 nm in *cenpa*(RNAi) and 423.1±30 nM in *mad-1* (p<0.0001). We also observed that there were fewer RAD-51 foci that were significantly larger in WT compared to *mad-1* and *cenpa*(RNAi) (number of RAD-51 foci: WT = 6.9±0.5, *mad-1* = 18.5±2.7, *cenpa*(RNAi) = 15.1±1.9; p<0.0001; area: WT = 0.11μm^2^, *mad-1* = 0.045μm^2^, *cenpa*(RNAi) = 0.053μm^2^; p<0.0001; Fig 6E). The larger patches of RAD-51 may represent the coalescence of several damage sites. Additionally, there was no significant difference between *mad-1* and *cenpa*(RNAi) when comparing either the distance of RAD-51 from the periphery, or the number and size of RAD-51 foci, suggesting MAD-1 and CENPA are both required to recruit damage to the periphery. Consistent with incorporation of CENPA in a subset of DNA, CENPA and DAPI did not show complete co-localization. Further, CENPA distribution was more diffuse in *mad-1* mutants by SIM (S5G Fig). Together, these data support a model whereby damaged DNA is brought to the nuclear periphery to mediate repair through interactions between CENPA and MAD-1 at nuclear pores.

### MAD2L1 is enriched in the nucleus in response to HU in human cells

**Fig 7:**
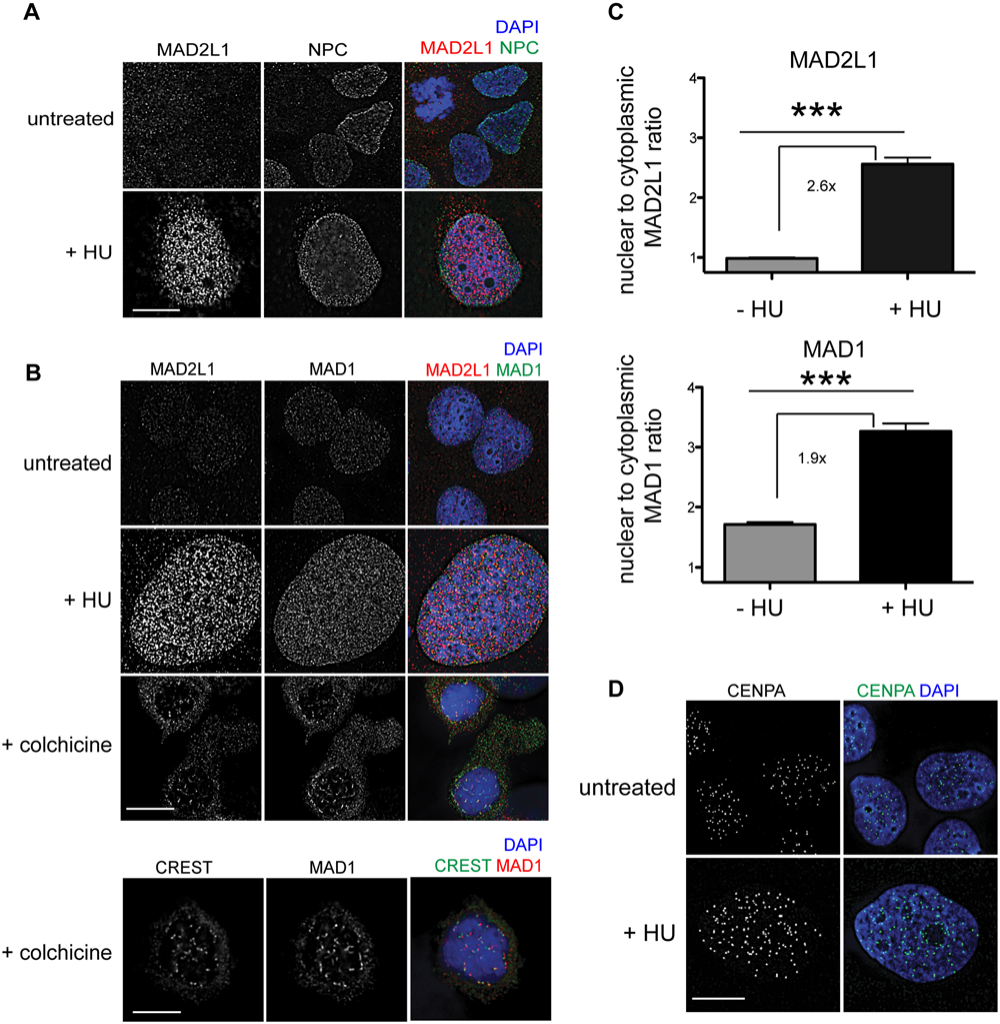
MAD1 and MAD2L1 are enriched in the nucleus in U2OS cells after HU exposure. (A) Untreated or HU treated U2OS cells stained with MAD2L1 (red) and Mab414 (NPC)(green) with DAPI (blue). (B) First panels show U2OS cells stained with MAD2L1 (red) or MAD1 (green) and counterstained with DAPI (blue) in untreated cells, with HU or with colchicine. Second panels show U2OS cells treated with colchicine and stained with CREST (green), MAD1 (red) and DAPI (blue). CREST recognizes CENP-A, -B, and—C. (C) Graph of the average ratio of nucleoplasmic MAD2L1 or MAD1 fluorescence to cytoplasmic signal in the presence and absence of HU; ***p<0.0001; n≥50; Error bars indicate SEM. (D) Untreated and HU treated U2OS cells stained with CENPA (green) and counterstained with DAPI (blue). Scale bars 10 μm.

To determine whether the SAC is engaged in response to DNA damage in mammals, we examined the localization of MAD1 and MAD2L1 after HU treatment in U2OS cells, a human osteosarcoma cell line. We found that similar to *C*. *elegans* germ cells, MAD2L1 was enriched in the nucleus 2.6 fold after HU treatment compared with no treatment (Fig 7A–7C). However, MAD2L1 appeared uniformly distributed in the nucleus and did not show further enrichment at the nuclear periphery (Fig 7A and 7B). In many organisms, MAD1 is detectable in the interphase nucleus with a population tethered at the nuclear pores [40,47–49], and we saw a similar localization in untreated cells (Fig 7B). After HU treatment, we observed enrichment of MAD1 in the nucleus (1.9 fold over untreated) similar to MAD2L1 (Fig 7C), suggesting MAD2L1 and MAD1 interact in response to DNA damage. Consistent with previous studies, MAD1 and MAD2L1 are both enriched at the kinetochore in the presence of the spindle poison colchicine (Fig 7B). We observed similar enrichment in the nucleus of these SAC components in the fibroblast-like COS cells after HU (S6 Fig).

Previous studies in mammalian cells have indicated that CENPA localizes to sites of DNA damage [44,45]. To determine whether CENPA became enriched in the nucleus after HU in U2OS cells, we monitored CENPA localization in the presence and absence of HU. While the overall number of CENPA foci was similar in the presence and absence of HU, the foci appeared larger following HU treatment (Fig 7D), suggesting that CENPA could be engaged in response to stalled/collapsed replication forks. Taken together, the relocalization of MAD1 and MAD2 and alteration of CENPA after HU suggests SAC components play a conserved role in response to DNA damage and could contribute to DNA repair, similar to what we observe in *C*. *elegans* germ cells.

## Discussion

**Fig 8:**
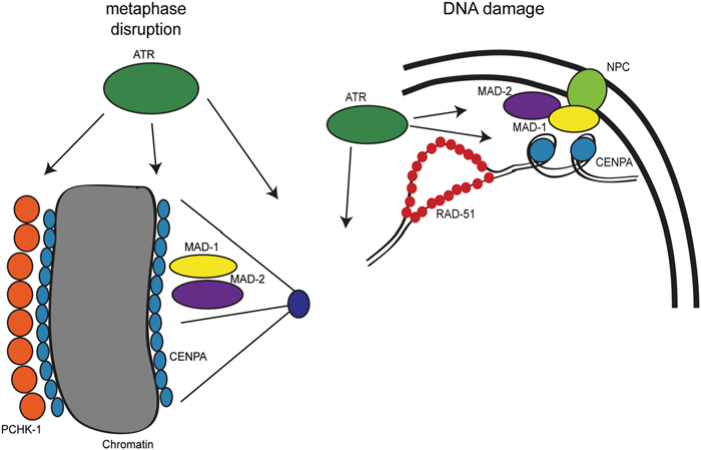
Model for DDR and SAC interactions throughout the cell cycle. During metaphase disruptions (left), ATR (green), P-CHK-1(Ser344) (orange), MAD-1 (yellow), MAD-2 (purple), and CENPA (blue) localize to chromatin and are required for metaphase delay and stable arrest. In response to DNA damage (right), RAD-51 (red), MAD-1, MAD-2, and CENPA localize to the nuclear periphery (NPC, light green). The localization of MAD-2 is dependent on ATR and MAD-1. Additionally, the localization of CENPA is dependent on ATR, MAD-1 and MAD-2. All of these components are required for efficient DNA repair.

We show here that the DDR and SAC function together at several points throughout the cell cycle in response to both DNA and spindle perturbations in *C*. *elegans* proliferating germ cells (Fig 8). Furthermore, we discovered a role for SAC components independent of CDC20 inhibition in facilitating both spindle stability and DNA repair. Our studies have implications for our understanding of checkpoint signaling, DNA repair, cell cycle control, and cancer chemotherapies.

### The role of the DDR in response to metaphase defects extends beyond CHK1

CHK1 plays a critical role in chromosome segregation; during unperturbed mitosis CHK1 localizes to kinetochores at metaphase [50–52], and depletion of CHK1 leads to chromosome misalignment and lagging chromosomes [51–53]. Further, CHK1 has been shown to be required for SAC-dependent metaphase arrest after taxol (microtubule stabilization) but not nocodazole (microtubule depolymerization) treatment in vertebrates [51,54]. Our studies reveal that CHK-1 plays a role once a bi-polar spindle has been assembled as it is required for DNA and spindle stability upon APC inactivation; however, in response to monopolar spindles (i.e., microtubule depolymerization), depletion of CHK-1 does not abrogate metaphase delay. In both cases, PCHK-1 Ser344, which is phosphorylated by ATM/ATR in response to DNA damage [32], accumulates along the length of metaphase chromosomes. These results suggest that both types of metaphase defects are sensed by upstream components of the DDR (Figs 1 and 8). Specifically, we have uncovered a role for ATR in both metaphase delay and stabilization thereby extending the scope of DDR function during metaphase. It is surprising that CHK-1 is phosphorylated in response to both monopolar spindle formation and following APC inactivation, yet is only required for the latter. One possibility is that other ATR substrates function either alone or redundantly with CHK-1 to delay the cell cycle in the presence of monopolar spindles. Alternatively, we are not achieving sufficient depletion of CHK-1; however, this would suggest that different thresholds of CHK-1 are required for metaphase delay versus stabilization of the metaphase plate.

### MAD-1 and MAD-2 are required for maintaining chromosome and spindle stability once chromosomes have bi-oriented

While evaluating metaphase arrest in an APC mutant, we discovered that stability of the metaphase plate was not only compromised when ATR or CHK-1 were depleted but also following inactivation of MAD-1 or MAD-2 (Figs 2 and 8). These results suggest that under these conditions, SAC function is independent of APC inhibition. Consistent with this, other components of the MCC, BUB-3 and MAD-3, were dispensable for metaphase plate stability. Thus, we propose that MAD-1 and MAD-2 play a novel role at the kinetochore independent of the MCC complex and APC inhibition. It is possible this role has remained undetected in mammalian cells because treatment with taxol or nocodazole leads to a metaphase delay not an arrest (H3S10P levels plateau around16hrs treatment [51,54]), in contrast to the stable metaphase arrest induced by the *C*. *elegans* APC mutant. Further, microtubule poisons, such as taxol and nocodazole, affect microtubule dynamics, suggesting that dynamic microtubules may be required for MAD-1 and MAD-2 signaling during persistent metaphase arrest. Finally, there is precedence for uncoupling the activation of MAD-1/MAD-2 and MAD-3/BUB-3 in the presence of monopolar spindles in the *C*. *elegans* embryo [27].

In mammalian cells, CHK1 functions through Aurora B to mediate spindle dynamics [51]. However, depletion of Aurora B/AIR-2 in *C*. *elegans* did not affect plate stability during arrest and localization of P-Aurora B was not altered by depletion of DDR or SAC, suggesting DDR and SAC do not mediate metaphase plates stability through regulation of Aurora B (S5F Fig). We propose that the DDR and SAC function together in response to metaphase defects, most likely through DDR phosphorylation of SAC components, as has been previously reported in high throughput screens and other studies [18,19,55–57]; however, the specific role of these phosphorylation events await future studies.

### DNA damage-induced enrichment of MAD-2 and histone variant CENPA at the nuclear periphery

While CHK1 has been previously shown to function during metaphase, to our knowledge we provide the first evidence that the SAC can be engaged in response to DNA damage at S and G2 independent of CDC20 inhibition. We show that in *C*. *elegans* proliferating germ cells, MAD-2 and CENPA become enriched at the nuclear periphery after DNA damage in a DDR-dependent manner. We propose that CENPA is incorporated into DNA after damage and interactions between CENPA and MAD-1-MAD-2 facilitate the translocation of damaged DNA to the nuclear periphery for repair (Fig 8). A DNA-damage-associated histone variant, similar to-H2AX in yeast and mammals, has yet to be identified in *C*. *elegans* [58], suggesting that CENPA could serve this role. However, CENPA is not enriched in meiotic cells, either in response to programmed meiotic DSBs or IR-induced breaks (S5H Fig). Perhaps the holocentric nature of *C elegans* chromosomes has driven the use of CENPA to serve a dual role in kinetochore function and DNA damage response specifically in the mitotic cell cycle; in meiosis, chromosomes are not holocentric as the kinetochore is defined by the site of crossing over independent of CENPA [59].

Interestingly, in mammalian cells, CENPA has been postulated to be involved in DNA damage signaling and repair independent of its kinetochore function [44,45,60]. Our data in *C*. *elegans* also supports a role for CENPA independent of centromere formation (Figs 6 and S5). Although we did not see observable changes in the number of CENPA foci or their localization after HU in human osteosarcoma cells, this could be a consequence of different types of damage (i.e. HU versus IR/laser) as we did see more robust recruitment of CENPA throughout the nucleus. Interestingly, many of the studies showing an association between CENPA and DNA damage have been performed in stem cells and *C elegans* proliferating germ cells have stem cell-like properties [61,62]. Thus, it is possible that stem cells regulate CENPA localization after DNA damage differently than somatic cells. In fact checkpoint responses in general may be different in stem cells as the cell cycle is altered [61,62] and it is critical to protect the genome in cells destined to contribute to all tissue in an organism. Nonetheless, the enrichment of SAC components MAD1 and MAD2 to the nucleus in human osteosarcoma cells suggests that the damage response mechanism we propose in *C*. *elegans* may be conserved in mammals.

### Nuclear pores as hubs for DNA repair

We found that RAD-51, CENPA, and MAD-2 localize to the nuclear periphery after DNA damage and that localization of CENPA and MAD-2 is dependent on ATR, MAD-1 and nuclear pore component NUP-107 (Figs 3, 5, 6 and S3). Further, RAD-51 foci are no longer closely associated with the nuclear periphery in the absence of MAD-1 or CENPA and depletion of CENPA, MAD-1 or MAD-2 renders germ cells incompetent for efficient DNA repair (Figs 4–6). We hypothesize that nuclear pores serve as a hub, analogous to the kinetochore, for recruiting damaged DNA and SAC and DDR components to facilitate efficient DNA repair (Fig 8). In *C*. *elegans*, translesion synthesis polymerases interact with nuclear pore components, and loss of these components leads to DNA damage sensitivity [63]. Similarly, loss of NUP107 in budding yeast leads to DNA damage sensitivity and is required for DNA damage localization to nuclear pores [64–66]. Additionally, a recent study found that recombination sites moved to the nuclear envelope for repair in Drosophila oocytes [67]. In mammalian cells a single DSB has been shown to remain stationary within the nucleus [68]; however, while the majority of the genome is not mobile following DNA damage certain chromosomes can move either inwardly or outwardly [69]. The restrained movement of some chromosomes in mammalian cells may explain why we don’t see nuclear peripheral enrichment of SAC components, MAD1 and MAD2, but instead see an even nuclear distribution. Perhaps CENPA, MAD1, and MAD2 are functioning in mammalian cells to facilitate DNA repair, but damage is not specifically recruited to the periphery due to increased genomic complexity.

### DDR and SAC in cancer

Consistent with the roles of both the SAC and DDR in protecting the genome, SAC and DDR mis-regulation has been documented in cancer [4,5,70]. Further, loss of DDR components ATM, ATR, or CHK1 lead to either embryonic lethality in mice and/or a predisposition for cancer [32,71–74]. Similarly, mice lacking MAD1, MAD2, BUBR1, or BUB3 do not survive past embryonic day 6.5–8.5; but heterozygous mice survive and have high rates of tumorigenesis [75–81]. Interestingly, a mouse line harboring a SAC-insensitive CDC20 allele survives longer during embryogenesis than SAC mutants [82], suggesting that SAC components function independently of APC inhibition, as we have shown in *C*. *elegans* germ cells.

Many cancer therapeutics are designed to induce DNA damage or metaphase defects, which activate the DDR or SAC to trigger apoptotic cell death (e.g., cisplatin, doxorubicin, paclitaxel) under the premise that these checkpoints are independent [83,84]. However, our studies indicate that loss of SAC or DDR components may compromise checkpoint function in response to both DNA damage and spindle perturbations and therefore have implications for cancer therapy success. Indeed, carcinoma cell lines with decreased MAD2 expression had decreased sensitivity to DNA crosslinking agent, cisplatin [85]. Our studies in *C*. *elegans* germ cells and human osteosarcoma cells underscore the importance of understanding the intersection between SAC and DDR in checkpoint signaling in response to both DNA and spindle perturbations.

## Materials and Methods

### Genetics

Strains were maintained at 20°C under standard conditions unless otherwise noted. Wild-type strain was N2 Bristol. Strains were obtained from the CGC unless otherwise noted. Strains used in this study: *san-1/mad-3(ok1580)* I, *atm-1(gk186) I*, *fzy-1(av15) unc-4(e120) II*, *npp-5/nup-107(tm3039)/mIn1 [mIs14 dpy-10(e128)] II*, *atl-1/ATR(tm853)* IV/nT1 [*unc-*?*(n754) let-*? *qIs50*] (IV;V), *mdf-1/mad-1(gk2)* V/nT1 [*unc-*?*(n754) let-*? *qIs50*] (IV;V), *fog-2(q71) V*, *atm-1(gk186)* I;*atl-1/ATR(tm853)* IV/nT1 [*unc-*?*(n754) let-*? *qIs50*] (IV;V). *mat-2(ax102)* II, and *zyg-1(b1)* II were maintained at 15°C. GFP::MAD-2 was a gift from Arshad Desai [27].

### Cytological analyses

Immunostaining of germ lines was performed as described in [34]. Germ lines were fixed in 1% PFA for 5 min, freeze-cracked in liquid nitrogen, followed by 1 min cold (-20°C) methanol. Slides were blocked in 0.7% BSA for 1 hr before primary antibodies were incubated at room temperature overnight. Secondary antibodies were incubated for 2 hrs at room temperature. Embryos were dissected on poly-lysine coated slides, freeze-cracked with liquid nitrogen, and fixed with cold (-20°C) methanol for 10 min. After air-drying, slides were rehydrated with 1X PBS followed by blocking in 0.7% BSA for 1 hr. Specificity of antibody staining was verified by examining the absence of staining in RNAi depleted or mutant worms.

The following primary antibodies were purchased and used at the indicated dilutions: rabbit anti-RAD-51 (1:10000), rabbit anti-GFP (1:500), rabbit anti-HCP-3 (1:500) rabbit anti-NCD-80 (1:500)(Novus Biologicals), P-CHK-1(Ser345)(1:50) (Santa Cruz Biotechnology), rabbit H3S10P (1:200)(Millipore), mouse anti-alpha tubulin (DM1α)(1:500)(Sigma Aldrich), mouse anti-nuclear pore complex proteins [Mab414](1:100)(abcam), rabbit anti-Aurora B Phospho Thr 232 (1:500)(Rockland Antibodies and Assays). Rabbit anti-MDF-1 (1:2000) [26], rabbit anti-MDF-2 (1:10000) [27], rabbit anti-SPD-2 (1:500) [36], and rat anti-RAD-51 (1:100) [86] were generous gifts from A. Desai, R. Kitagawa, K. Oegema, and A. Villeneuve, respectively. The following secondary antibodies from Life Technologies were used at 1:500 dilutions: Alexa Fluor 555 goat anti-rabbit IgG, Alexa Fluor 488 goat anti-rabbit IgG, Alexa Fluor 546 goat anti-mouse IgG, Alexa Fluor 488 goat anti-mouse IgG. Alexa Fluor 647 donkey anti-mouse IgG was used at a 1:200 dilution. DAPI (2 μg/ml; Sigma) was used to counterstain DNA.

Collection of images was performed using an API Delta Vision deconvolution microscope. Images were deconvolved using Applied Precision SoftWoRx image analysis software and were subsequently processed and analyzed using Fiji (ImageJ) (Wayne Rasband, NIH). All images are projections through approximately half of the germ line unless otherwise stated.

Structured illumination microscopy (SIM) analysis was performed using a Nikon N-SIM super-resolution microscope and NIS-Elements 2 image processing software. Images were further processed using ImageJ.

### CENPA intensity

L4s were treated with 0 or 25mM HU for 16 hrs and allowed to recover for 5 hrs before dissection and staining with CENPA and Mab414 (NPC). Germ lines were imaged at the same exposure time for CENPA and the CENPA channel was not manipulated post-acquisition. To determine fluorescence intensity, a single z stack was chosen in which the middle of several nuclei were displayed. A line was drawn across a single nucleus and the RGB plot profile was collected in Image J. Intensities were binned and averaged in 10% increments of nuclear length. Measurements were taken for every arrested (enlarged) nucleus where the plane bisected the middle of the nucleus for 3 germ lines per condition and these measurements were averaged.

### RAD-51 measurements in SIM images

Distances between RAD-51 and NPC were determined by obtaining fluorescence intensity plots with line scans in Image J. The number of pixels between the peaks of each signal was determined and converted to nanometers.

Statistics were determined with an unpaired student’s T-test or two-way ANOVA.

### RNA-mediated interference (RNAi) analysis

RNAi experiments were performed using the feeding method [87] at 20°C, except for experiments using *mat-2(ax102)*and *zyg-1(b1)*, which were propagated at 15°C. Unless otherwise noted, gravid hermaphrodites were fed RNAi-inducing HT115(DE3) bacteria strains or the same bacteria transformed with the empty feeding vector, L4440. *chk-1*(RNAi) was performed on L1 larvae. All feeding strains were obtained from a genomic RNAi feeding library [88]. Cultures were plated onto NGM plates containing 25μg/ml Carbenicillin and 1mM IPTG and were used within two weeks.

### Assessment of SAC RNAi efficiency

Following depletion of SAC, L4 worms were fed *cyb-3* RNAi for 24 hrs. Worms were dissected and embryos were placed on a 3% agarose pad and imaged by DIC at 40X on a Ziess compound microscope to monitor arrest.

### Irradiation experiments

Young adult worms were irradiated with 30Gy (3000 rad) from a Cs-137 source. Worms were dissected 8 hrs post irradiation for MAD-2 and CENPA localization studies and 24 hrs post irradiation for recovery experiments.

### Hydroxyurea experiments

For high dose experiments, L4s were placed on NGM plates containing 25mM hydroxyurea (HU) (Sigma Aldrich) for 16 hrs before either dissection, transfer for recovery, or progeny viability assays. Cell cycle arrest was assayed by counting DAPI stained nuclei for *chk-1* and *atr* RNAi efficiency. For low dose HU experiments, staged young adults were exposed to 5mM HU for 2 hrs before being moved to a—HU plate for dissection, recovery, or progeny viability assays. HU was allowed to dissipate into plates for at least 3 hrs before worms were introduced. For low dose HU exposure, cell cycle kinetics were assayed by counting H3S10P.

### Metaphase arrest and delay

For antibody staining after MAT-2 or ZYG-1 inactivation, *mat-2(ax102)* and *zyg-1(b1)* L4s were transferred to the restrictive temperature of 25°C for 16 or 48 hrs before dissection, respectively. To determine metaphase delay/arrest, *zyg-1(b1)* and *mat-2(ts)* L4s were transferred to the restrictive temperature of 25°C for 24 hrs before dissection and staining with H3S10P.

### Western

Worm lysates were generated from unmated *fog-2(q71)* worms to eliminate contribution from embryos and were resolved on 12% SDS-PAGE gels and transferred to Millipore Immobilon-P PVDF membranes. Membranes were blocked with 5% BSA and probed with rabbit anti-CENPA (1:250, Novus), and anti-Mortalin/Grp75 mouse monoclonal antibody N52A/42 (1:20, AB_10674108; UC Davis/NIH NeuroMab Facility, Davis, CA) as loading control., followed by IRDye680LT- and IRDye800-conjugated anti-rabbit and anti-mouse IgG secondary antibodies obtained from LI-COR Bioscience (Lincoln, NE). 0.01% SDS was added to anti-rabbit secondary.

### Cell lines

Human U2OS osteosarcoma cells and COS monkey kidney cells were, obtained from the ATCC. U2OS cells were grown in McCoy’s 5A modified medium, COS cells were grown in DMEM and both were supplemented with 10% fetal bovine serum and were cultured at 37°C in 5% CO_2_.

### Immunofluorescence in cell lines

Cells were grown on glass coverslips and treated with 5mM HU for 24 hrs or 1μg/mL colchicine (Sigma) for 16 hrs. Cells were fixed with 4% paraformaldehyde and 0.1% triton, then blocked with 5% BSA for 1 hr before primary antibodies were added and incubated at room temperature overnight. Secondary antibodies were incubated for 2 hrs at room temperature.

To determine fluorescence intensity, integrated density was identified for 2 equal areas in both the nucleus and the cytoplasm. The ratio of nuclear to cytoplasmic signal was calculated dividing the averages of the 2 measurements. For each condition, n≥50 cells.

Primary antibodies were used at the following dilutions: rabbit anti-H3S10P (1:500) (Millipore), rabbit anti-MAD2L1 (1:500) and mouse anti-nuclear pore complex (MAb414) (1:500)(Abcam), mouse anti-CENPA (1:100)(GeneTex), mouse anti-MAD1L1 IgG2b (1:250)(Acris Antibodies, Inc) and human CREST antiserum (1:500) (a generous gift from N. Hunter [89]). The following secondary antibodies from Life Technologies were all used at 1:2000 dilutions: Alexa Fluor 555 goat anti-rabbit IgG, Alexa Fluor 488 goat anti-mouse IgG1 and IgG2b, Molecular Probes anti-human 488, 1:2000. DAPI (2μg/ml; Sigma) was used to counterstain DNA.

## Supporting Information

S1 Fig SAC and kinetochore localization in proliferating germ cells and in embryos. A) Metaphase arrested nuclei from *mat-2(ts)* worms stained for either CENPA (red) or both CENPA and MAD-1 (red) and co-stained with α-tubulin (green) and DAPI (blue). The average width of CENPA staining is 308±12nM, the average width of CENPA/MAD-1 staining is 316±14nM, p = 0.87 (B) Nucleus with a monopolar spindle from a *zyg-1(ts)* worm stained with NDC-80 (red) α-tubulin (green) and DAPI (blue). Scale bars = 2mM. (C) Wild-type and GFP::MAD-2 embryos either arrested at 1-cell stage by *cyb-3*(RNAi) or not arrested and stained for either MAD-1 or MAD-2 (red), α-tubulin (green) and DAPI (blue). Arrows indicate MAD-1 or MAD-2 staining. (D) DAPI stained germ lines of wild type and *chk-1(*RNAi) worms with and without HU. Scale bars = 10μM.
(TIF)

S2 Fig Neither DDR nor SAC mutants display aberrant spindle or DNA morphology in the absence of metaphase arrest. (A) Dissected germ lines from *atr(tm853)*, *chk-1*(RNAi), *mad-1*(RNAi) and *mad-3*(RNAi) and *mat-2(ts);mad-2*(RNAi) treated worms at 25° stained with H3S10P (red), α-tubulin (green) and DAPI (blue). (B) Percentage of tubulin arrays in proliferative zones of the above genotypes at 25° (n≥10). (C) Efficiency of SAC RNAi as measured by failure to arrest in the early embryo following *cyb-3*(RNAi). Percentage of cell numbers in the early embryo of the given genotypes (n≥100).
(TIF)

S3 Fig Requirements for MAD-1 and MAD-2 localization in response to DNA damage. (A) MAD-2 is not enriched at the nuclear periphery after cell cycle disruption. Depletion of CDK-2 or CYE-1by RNAi does not induce MAD-2 (red) enrichment to the nuclear periphery (NPC, green), however germ lines are competent for MAD-2 relocalization if treated with HU. (B) MAD-2 is not enriched at the nuclear periphery in *nup-107*. In the presence of HU, MAD-1 (red) and MAD-2 (red) fail to localize to the nuclear periphery (NPC, green) in *nup-107(tm3039)*. (C) MAD-1(green) and DAPI (magenta) in the proliferative zones of wild-type and *atr(tm853)* germ lines in the presence and absence of HU. Scale bar = 10μm.
(TIF)

S4 Fig Requirement for SAC components in response to DNA damage in germ line proliferating germ cells. (A) Percent of nuclei that contain at least 1 RAD-51 focus—HU or after 0, 2, 4, 6, or 16 hours of 5mM HU recovery for wild-type, *mad-1(gk2)*, *atr(tm853)*, and *fzy-1(av15)* worms (n>10). (B) Cartoon of the germ line showing approximately how long it takes for nuclei to travel through the germ line to form embryos. (C) Progeny inviability after 5mM HU separated into embryos laid in the 1^st^ 24 hrs and the last 48 hrs for wild type and *mad-3(ok1580)* (n>13) (D) Dissected WT germ line showing MAD-2 (green) accumulation in nuclei after release from 5mM HU treatment, DAPI (magenta). Scale bar = 10μm. (E) Percent of nuclei with RAD-51 in wild type *fzy-1(av15)*, *mad-3(ok1580)*, *mad-1(gk2)*, and *atr(tm853)* germ lines after recovery from 30 gy of gamma irradiation. *p<0.05, ***p<0.0001 (two-way ANOVA).
(TIF)

S5 Fig CENPA but not NDC-80 is enriched in the nucleus following DNA damage. (A) Proliferative zones of wild-type worms after IR or in the absence of damage stained with CENPA (red) and DAPI (blue). (B) CENPA steady state levels are not up-regulated after HU. Western blot showing CENPA in *fog-2(q71)* worms with and without HU treatment and in worms depleted for CENPA. Mortalin was used as a loading control. (C) NDC-80 is not enriched in the nucleus after HU. Wild-type germ lines stained with NDC-80 (red) and DAPI (blue) in the presence and absence of HU. (D) Partial depletion of CENPA by *cenpa*(RNAi). Germ line stained with CENPA (red) and DAPI (blue). (E) *atr(tm853)* worms are still competent for loading CENPA during metaphase. *atr(tm853)* germ line stained for CENPA (red) and DAPI (blue). Arrows indicate CENPA staining. Scale bars = 10μm. (F) P-AIR-2 localization is not disrupted after depletion of DDR or SAC in metaphase arrested nuclei. P-AIR-2(red), α-tubulin (green) and DAPI (blue) staining in *mat-2(ts)*, *mat-2(ts);atr*(RNAi), and *mat-2(ts);mad-1*(RNAi) germ lines. (G) SIM images of nuclei from wild type and worms treated with HU and stained for CENPA(cyan), DAPI(magenta), and NPC(yellow). Scale bar 2 μm. (H) CENPA is not enriched in meiotic nuclei. Germ line from an HU-treated wild-type worm stained with CENPA (red) and DAPI (blue). Arrows indicate pachytene nuclei. Scale bar = 10μm.
(TIF)

S6 Fig MAD2L1 is enriched in the nucleus in COS cells after HU exposure. (A) COS cells stained with MAD2L1 (red) or MAD1 (green) and counterstained with DAPI (blue) in untreated cells, with colchicine or HU. (B) Graph shows the average ratio of nucleoplasmic MAD2L1 fluorescence to cytoplasmic signal in the presence and absence of HU; Error bars indicate SEM. Scale bar = 2μm.
(TIF)
