# Supplementary figures and images for: DNA Damage Response and Spindle Assembly Checkpoint Function throughout the Cell Cycle to Ensure Genomic Integrity
Source: PLoS Genet. 2015 Apr 21;11(4):e1005150. doi: 10.1371/journal.pgen.1005150 (PMC4405263; doi:10.1371/journal.pgen.1005150)

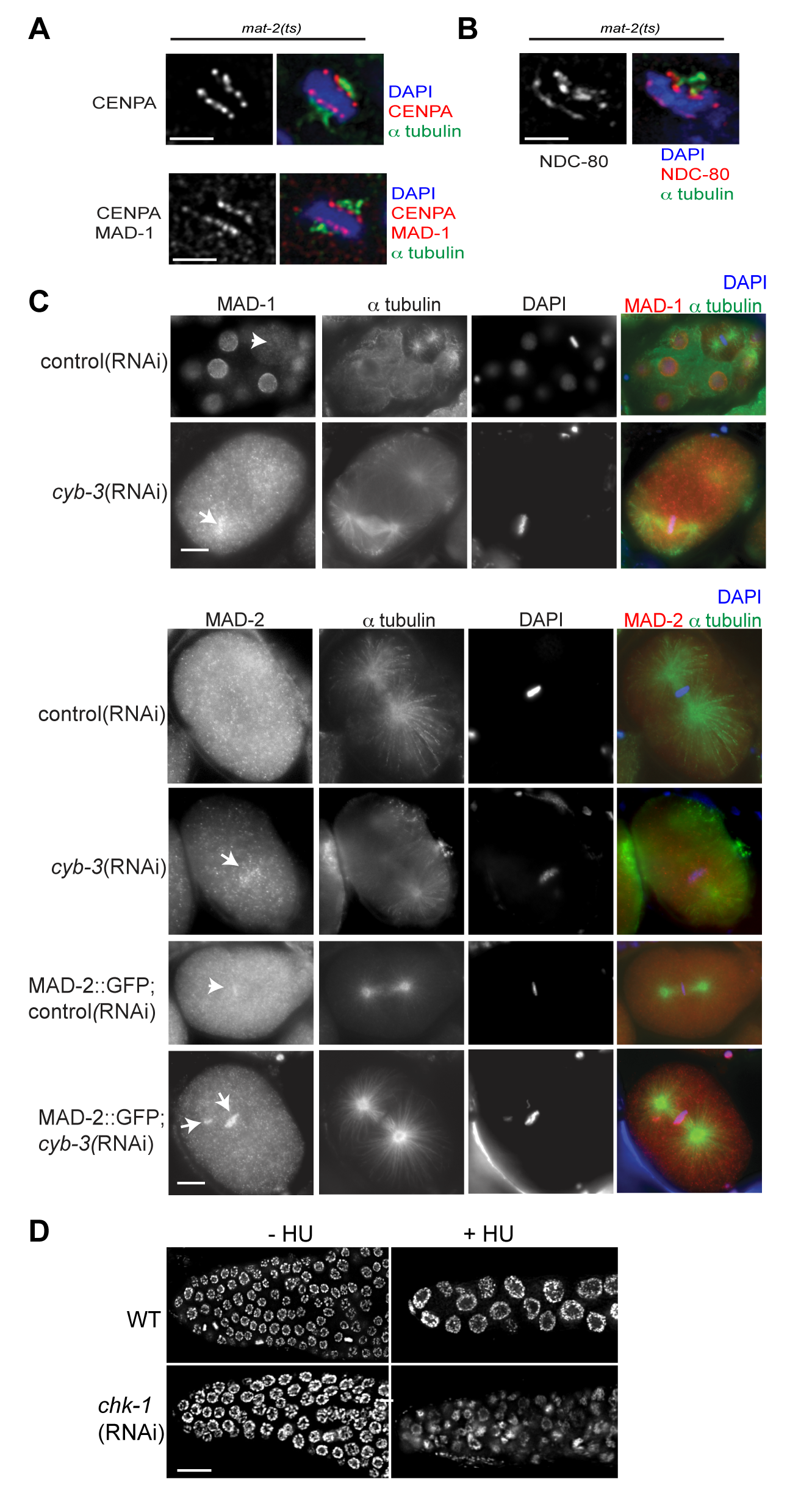

Supplement: S1 Fig — A) Metaphase arrested nuclei from mat-2(ts) worms stained for either CENPA (red) or both CENPA and MAD-1 (red) and co-stained with α-tubulin (green) and DAPI (blue). The average width of CENPA staining is 308±12nM, the average width of CENPA/MAD-1 staining is 316±14nM, p = 0.87 (B) Nucleus with a monopolar spindle from a zyg-1(ts) worm stained with NDC-80 (red) α-tubulin (green) and DAPI (blue). Scale bars = 2mM. (C) Wild-type and GFP::MAD-2 embryos either arrested at 1-cell stage by cyb-3(RNAi) or not arrested and stained for either MAD-1 or MAD-2 (red), α-tubulin (green) and DAPI (blue). Arrows indicate MAD-1 or MAD-2 staining. (D) DAPI stained germ lines of wild type and chk-1(RNAi) worms with and without HU. Scale bars = 10μM. (TIF) [file pgen.1005150.s001.tif]

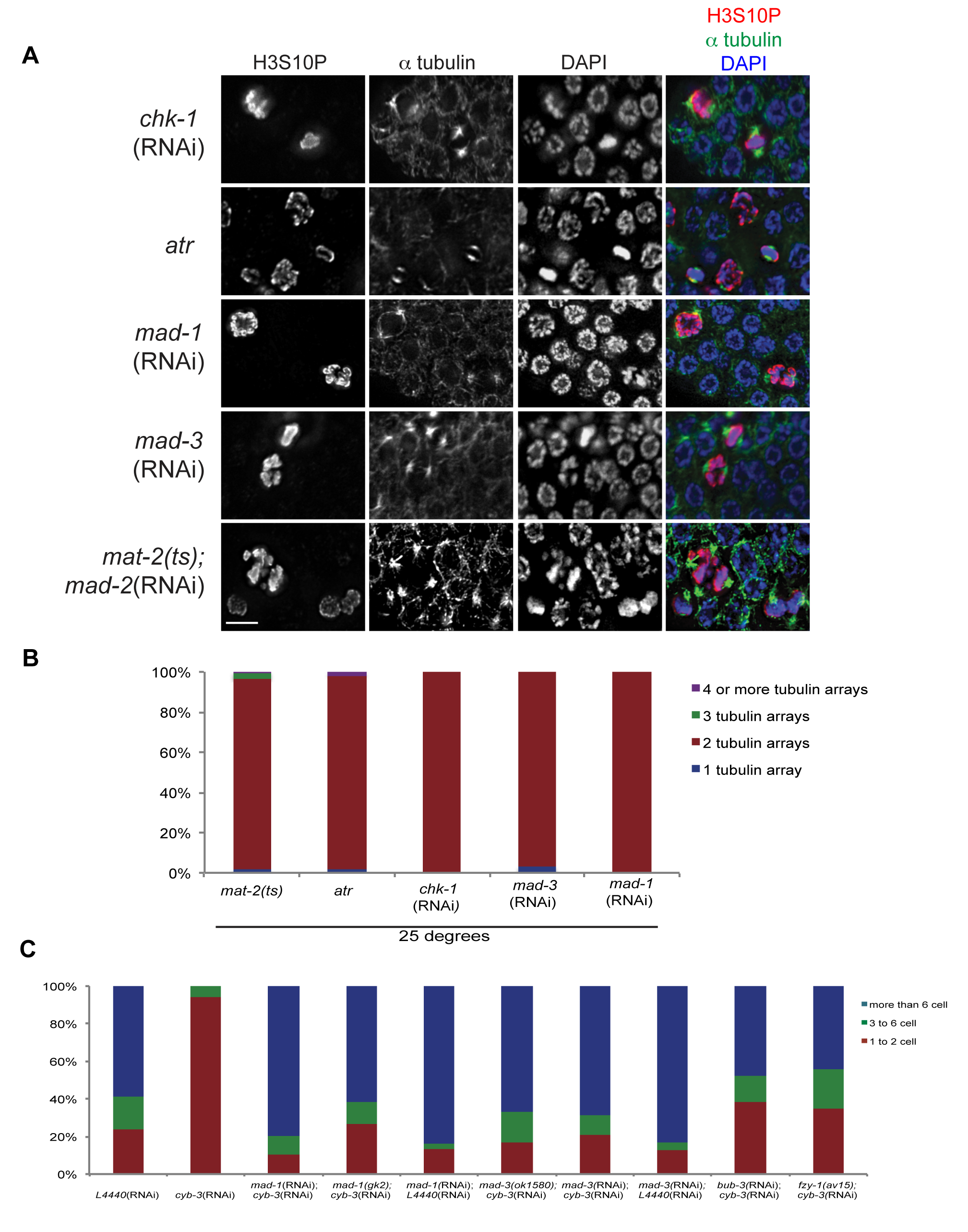

Supplement: S2 Fig — (A) Dissected germ lines from atr(tm853), chk-1(RNAi), mad-1(RNAi) and mad-3(RNAi) and mat-2(ts);mad-2(RNAi) treated worms at 25° stained with H3S10P (red), α-tubulin (green) and DAPI (blue). (B) Percentage of tubulin arrays in proliferative zones of the above genotypes at 25° (n≥10). (C) Efficiency of SAC RNAi as measured by failure to arrest in the early embryo following cyb-3(RNAi). Percentage of cell numbers in the early embryo of the given genotypes (n≥100). (TIF) [file pgen.1005150.s002.tif]

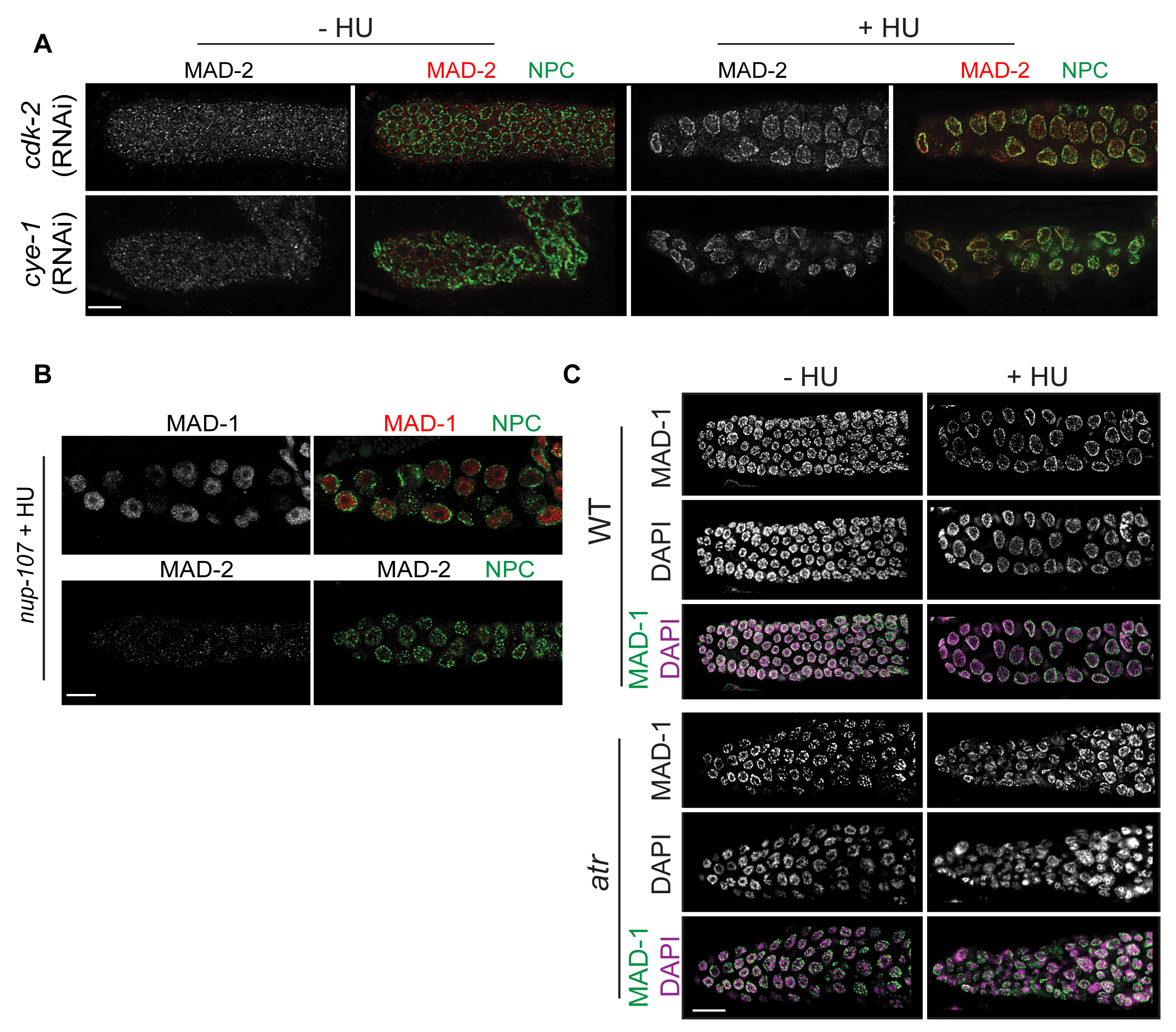

Supplement: S3 Fig — (A) MAD-2 is not enriched at the nuclear periphery after cell cycle disruption. Depletion of CDK-2 or CYE-1by RNAi does not induce MAD-2 (red) enrichment to the nuclear periphery (NPC, green), however germ lines are competent for MAD-2 relocalization if treated with HU. (B) MAD-2 is not enriched at the nuclear periphery in nup-107. In the presence of HU, MAD-1 (red) and MAD-2 (red) fail to localize to the nuclear periphery (NPC, green) in nup-107(tm3039). (C) MAD-1(green) and DAPI (magenta) in the proliferative zones of wild-type and atr(tm853) germ lines in the presence and absence of HU. Scale bar = 10μm. (TIF) [file pgen.1005150.s003.tif]

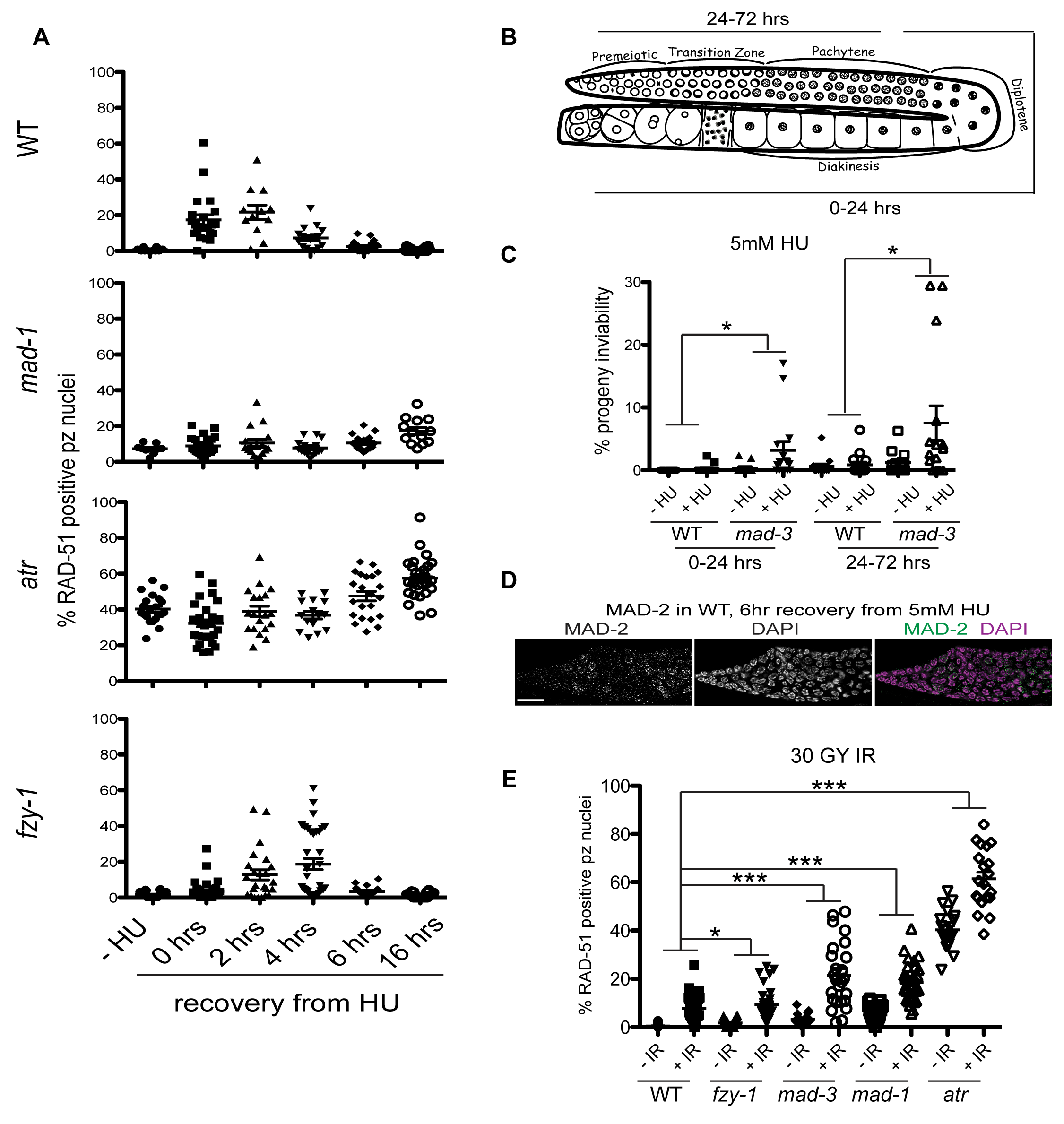

Supplement: S4 Fig — (A) Percent of nuclei that contain at least 1 RAD-51 focus—HU or after 0, 2, 4, 6, or 16 hours of 5mM HU recovery for wild-type, mad-1(gk2), atr(tm853), and fzy-1(av15) worms (n>10). (B) Cartoon of the germ line showing approximately how long it takes for nuclei to travel through the germ line to form embryos. (C) Progeny inviability after 5mM HU separated into embryos laid in the 1st 24 hrs and the last 48 hrs for wild type and mad-3(ok1580) (n>13) (D) Dissected WT germ line showing MAD-2 (green) accumulation in nuclei after release from 5mM HU treatment, DAPI (magenta). Scale bar = 10μm. (E) Percent of nuclei with RAD-51 in wild type fzy-1(av15), mad-3(ok1580), mad-1(gk2), and atr(tm853) germ lines after recovery from 30 gy of gamma irradiation. *p<0.05, ***p<0.0001 (two-way ANOVA). (TIF) [file pgen.1005150.s004.tif]

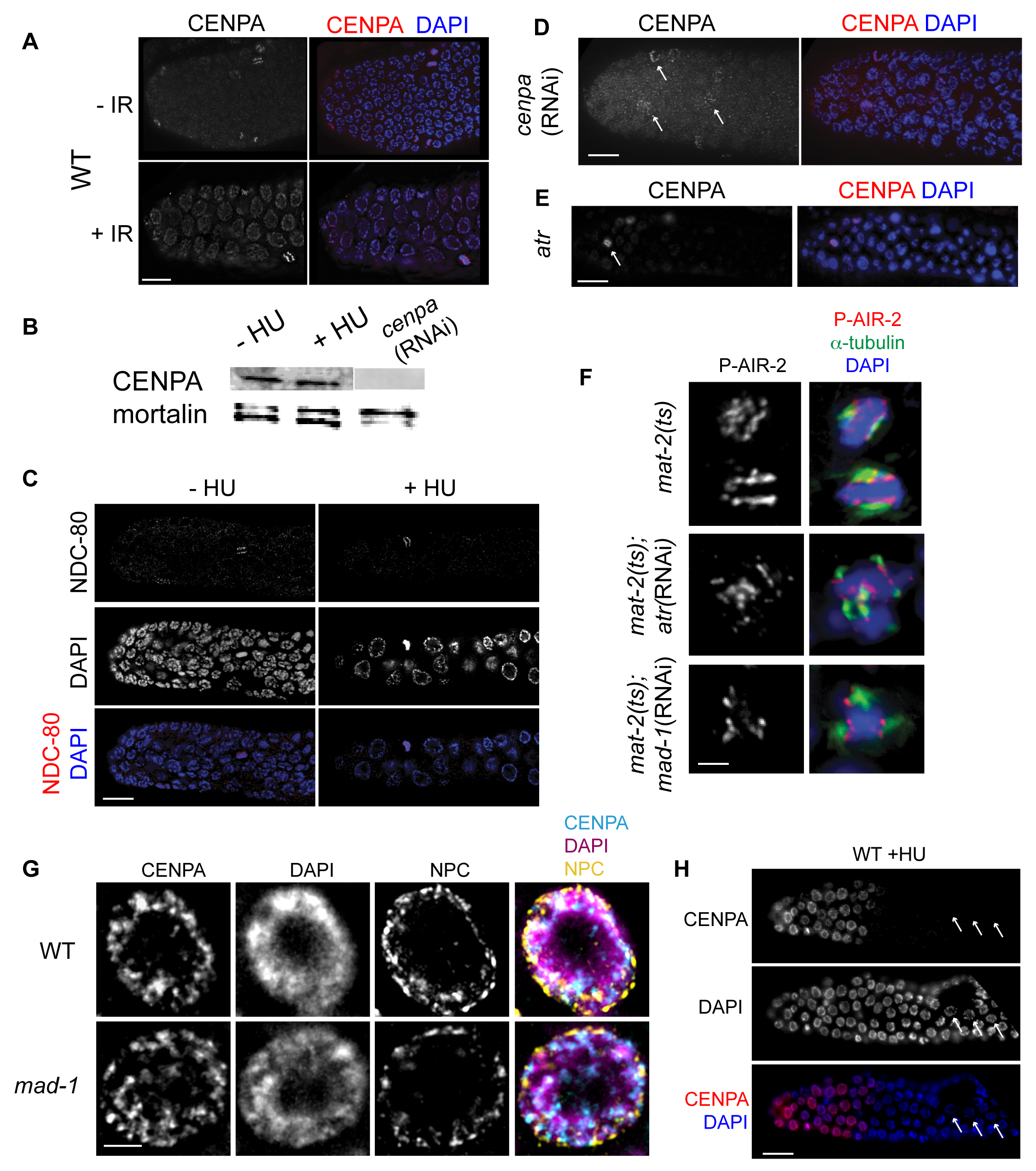

Supplement: S5 Fig — (A) Proliferative zones of wild-type worms after IR or in the absence of damage stained with CENPA (red) and DAPI (blue). (B) CENPA steady state levels are not up-regulated after HU. Western blot showing CENPA in fog-2(q71) worms with and without HU treatment and in worms depleted for CENPA. Mortalin was used as a loading control. (C) NDC-80 is not enriched in the nucleus after HU. Wild-type germ lines stained with NDC-80 (red) and DAPI (blue) in the presence and absence of HU. (D) Partial depletion of CENPA by cenpa(RNAi). Germ line stained with CENPA (red) and DAPI (blue). (E) atr(tm853) worms are still competent for loading CENPA during metaphase. atr(tm853) germ line stained for CENPA (red) and DAPI (blue). Arrows indicate CENPA staining. Scale bars = 10μm. (F) P-AIR-2 localization is not disrupted after depletion of DDR or SAC in metaphase arrested nuclei. P-AIR-2(red), α-tubulin (green) and DAPI (blue) staining in mat-2(ts), mat-2(ts);atr(RNAi), and mat-2(ts);mad-1(RNAi) germ lines. (G) SIM images of nuclei from wild type and worms treated with HU and stained for CENPA(cyan), DAPI(magenta), and NPC(yellow). Scale bar 2 μm. (H) CENPA is not enriched in meiotic nuclei. Germ line from an HU-treated wild-type worm stained with CENPA (red) and DAPI (blue). Arrows indicate pachytene nuclei. Scale bar = 10μm. (TIF) [file pgen.1005150.s005.tif]

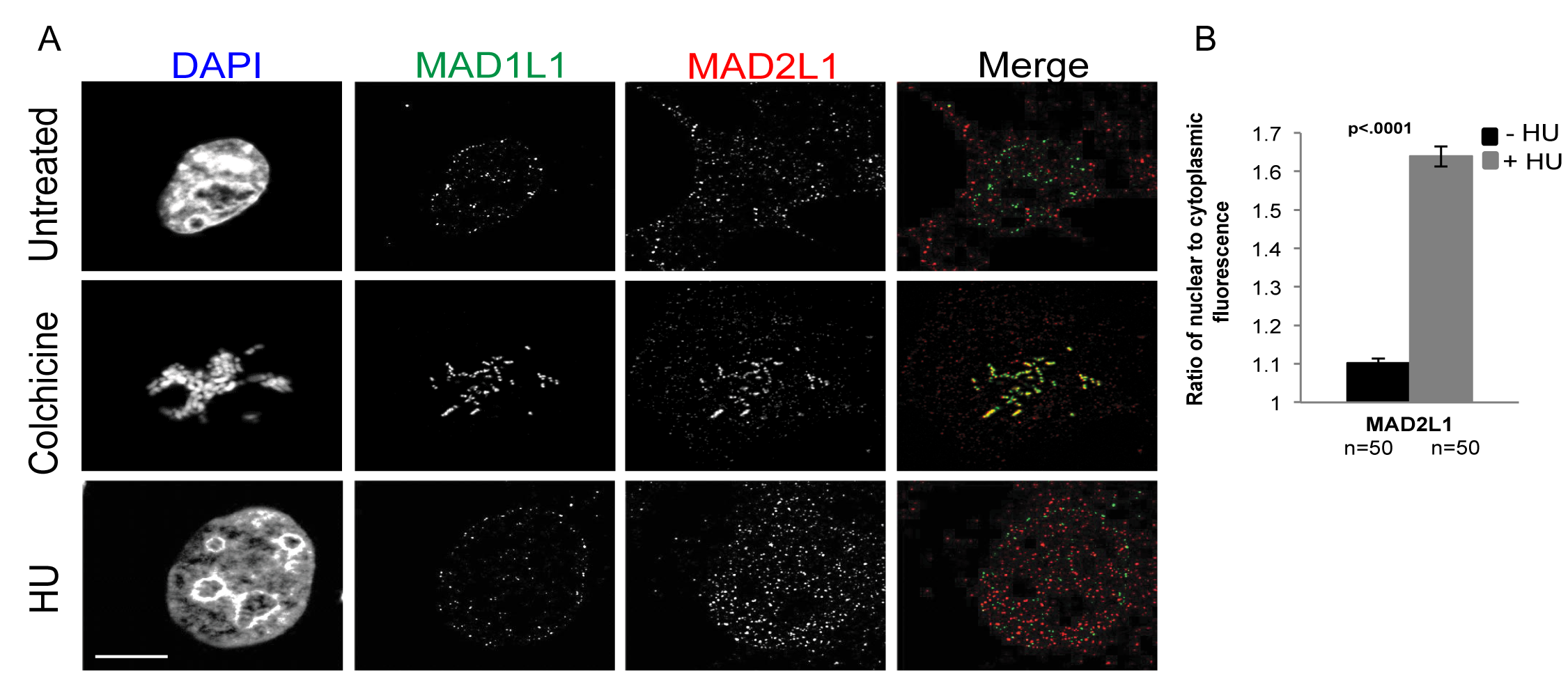

Supplement: S6 Fig — (A) COS cells stained with MAD2L1 (red) or MAD1 (green) and counterstained with DAPI (blue) in untreated cells, with colchicine or HU. (B) Graph shows the average ratio of nucleoplasmic MAD2L1 fluorescence to cytoplasmic signal in the presence and absence of HU; Error bars indicate SEM. Scale bar = 2μm. (TIF) [file pgen.1005150.s006.tif]
